# Survival‐Assured Liver Injury Preconditioning (SALIC) Enables Robust Expansion of Human Hepatocytes in *Fah* ^–/–^ *Rag2* ^–/–^ *IL2rg* ^–/–^ Rats

**DOI:** 10.1002/advs.202101188

**Published:** 2021-08-11

**Authors:** Ludi Zhang, Jian‐Yun Ge, Yun‐Wen Zheng, Zhen Sun, Chenhua Wang, Zhaoliang Peng, Baihua Wu, Mei Fang, Kinji Furuya, Xiaolong Ma, Yanjiao Shao, Nobuhiro Ohkohchi, Tatsuya Oda, Jianglin Fan, Guoyu Pan, Dali Li, Lijian Hui

**Affiliations:** ^1^ State Key Laboratory of Cell Biology, Shanghai Institute of Biochemistry and Cell Biology, Center for Excellence in Molecular Cell Science, Chinese Academy of Sciences University of Chinese Academy of Science Shanghai 200031 China; ^2^ Department of Gastrointestinal and Hepato‐Biliary‐Pancreatic Surgery, Faculty of Medicine University of Tsukuba Tsukuba Ibaraki 305‐8575 Japan; ^3^ Guangdong Provincial Key Laboratory of Large Animal Models for Biomedicine School of Biotechnology and Heath Sciences Wuyi University Jiangmen Guangdong 529020 China; ^4^ Institute of Regenerative Medicine Affiliated Hospital of Jiangsu University Jiangsu University Zhenjiang Jiangsu 212001 China; ^5^ Yokohama City University School of Medicine Yokohama Kanagawa 234‐0006 Japan; ^6^ Shanghai Institute of Materia Medica Chinese Academy of Sciences Shanghai 201203 China; ^7^ Shanghai Key Laboratory of Regulatory Biology, Institute of Biomedical Sciences and School of Life Sciences East China Normal University Shanghai 200241 China; ^8^ Department of Molecular Pathology, Faculty of Medicine Interdisciplinary Graduate School of Medicine University of Yamanashi Shimokato Yamanashi 409‐3898 Japan; ^9^ School of Life Science and Technology ShanghaiTech University Shanghai 201210 China; ^10^ School of Life Science, Hangzhou Institute for Advanced Study University of Chinese Academy of Sciences Hangzhou 310024 China; ^11^ Institute for Stem Cell and Regeneration Chinese Academy of Sciences Beijing 100101 China; ^12^ Bio‐Research Innovation Center Shanghai Institute of Biochemistry and Cell Biology Suzhou Jiangsu 215121 China

**Keywords:** bioreactor, humanized liver, liver xeno‐repopulation, pharmacological study

## Abstract

Although liver‐humanized animals are desirable tools for drug development and expansion of human hepatocytes in large quantities, their development is restricted to mice. In animals larger than mice, a precondition for efficient liver humanization remains preliminary because of different xeno‐repopulation kinetics in livers of larger sizes. Since rats are ten times larger than mice and widely used in pharmacological studies, liver‐humanized rats are more preferable. Here, *Fah^–/–^Rag2^–/–^IL2rg^–/–^* (FRG) rats are generated by CRISPR/Cas9, showing accelerated liver failure and lagged liver xeno‐repopulation compared to FRG mice. A survival‐assured liver injury preconditioning (SALIC) protocol, which consists of retrorsine pretreatment and cycling 2‐(2‐nitro‐4‐trifluoromethylbenzoyl)‐1,3‐cyclohexanedione (NTBC) administration by defined concentrations and time intervals, is developed to reduce the mortality of FRG rats and induce a regenerative microenvironment for xeno‐repopulation. Human hepatocyte repopulation is boosted to 31 ± 4% in rat livers at 7 months after transplantation, equivalent to approximately a 1200‐fold expansion. Human liver features of transcriptome and zonation are reproduced in humanized rats. Remarkably, they provide sufficient samples for the pharmacokinetic profiling of human‐specific metabolites. This model is thus preferred for pharmacological studies and human hepatocyte production. SALIC may also be informative to hepatocyte transplantation in other large‐sized species.

## Graphical abstract

Liver‐humanized animals are the state‐of‐the‐art model systems to mimic both physiological and pathological features of human livers. Herein a survival‐assured liver injury preconditioning (SALIC) protocol is developed to stably establish humanized livers in *Fah^−/−^Rag2^−/−^IL2rg^−/−^* rats. This model is preferred for pharmacological studies and expansion of human hepatocytes. SALIC may also be informative to liver humanization in other large‐sized animals.

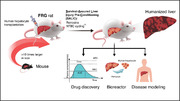

## 1 Introduction

The liver is a vital organ for the metabolism and clearance of drugs. Complete features of the liver in a particular species cannot be fully recapitulated in any other species, mainly reflected from their metabolism specificities. Humanized liver in mice was thus successfully achieved by transplanting primary human hepatocytes (PHHs) to livers of immunodeficient mice after xeno‐repopulation induced by liver injury.^[^ ^1^, ^2^ ^]^ As a model system, this mouse model with humanized liver was applied on human drug metabolism and hepatitis virus infection.^[^ ^3^ ^]^ In addition, liver xeno‐repopulation in immunodeficient mice was considered for in vivo expansion of human hepatocytes to be used for regenerative medicine.^[^ ^3^, ^4^ ^]^ It is noteworthy that liver‐humanized animals with a human hepatocyte xeno‐repopulation level >30% are required for most applications.^[^ ^5^ ^]^ The high level of human hepatocyte chimerism in mice, achieved through decades of methodological improvements, leads to many expectations for future pharmacological and clinical applications.^[^ ^6^, ^7^, ^8^, ^9^ ^]^ However, because of size limitations, mice cannot provide sufficient amounts of biological samples for pharmacological analyses or human hepatocytes for potential regenerative medicines.^[^ ^10^ ^]^ Hence, animals larger than mice, such as rats and pigs, have been actively studied to generate new models of humanized livers.^[^ ^3^ ^]^

Compared with mice, rats are at least ten times larger in size. In theory, one rat liver can produce up to 1 billion human hepatocytes if xeno‐repopulation is complete in the liver. Rats are also similar to humans in terms of many physiological and pathological aspects.^[^ ^11^ ^]^ They are recommended as the first choice for studying drug metabolism and toxicology.^[^ ^12^ ^]^ Importantly, many conclusive decisions are based solely on pharmacokinetic analyses using rats during drug discovery.^[^ ^13^ ^]^ On the other hand, when compared with pigs, rats have the advantage to be bred into severe immunodeficiency for xenotransplantation.^[^ ^14^ ^]^

Recent advances in the liver humanization of rats have not yet met the expectations for pharmacological and clinical applications. A major roadblock is the low efficiency of xeno‐repopulation of transplanted human hepatocytes in recipient rats. To achieve high xeno‐repopulation, it is necessary to develop an optimized precondition for human hepatocyte transplantation in rats. Previously, preconditioning with retrorsine, a pyrrolizidine alkaloid that specifically inhibits mitosis of rat hepatocytes, enabled the engraftment of PHHs in immunosuppressed rats. However, retrorsine pretreatment alone only led to scattered repopulation below 1%.^[^ ^15^ ^]^ In our recent study, the combination of retrorsine pretreatment and partial hepatectomy promoted xeno‐repopulation in *Rag1* ^–/–^ rats.^[^ ^16^ ^]^ Nevertheless, in all reported studies, the xeno‐repopulation of human hepatocytes has not reached 5% using retrorsine‐based preconditions.^[^ ^17^ ^]^

Fumarylacetoacetate hydrolase (Fah)‐deficiency, a model of tyrosinemia Type I, has been demonstrated as one of the most successful preconditions in mice, in which liver injury was induced by withdrawing the protective drug 2‐(2‐nitro‐4‐trifluoromethylbenzoyl)‐1,3‐cyclohexanedione (NTBC).^[^ ^18^ ^]^ We and other groups had generated a rat model of *Fah* gene knockout (*Fah^–/–^*),^[^ ^19^, ^20^ ^]^ in which NTBC withdrawal‐induced liver injury drove repopulation to a level up to 90% after syngeneic transplantation of wild‐type rat hepatocytes.^[^ ^19^ ^]^ However, with complete NTBC withdrawal, 40% *Fah^–/–^* rat recipients died after hepatocyte transplantation because of acute liver failure.^[^ ^19^ ^]^ By contrast, almost all *Fah^–/–^* mouse recipients survived after transplantation of syngeneic hepatocytes.^[^ ^21^ ^]^ These findings highlighted a remarkable difference in injury responses between *Fah*‐deficient rats and mice. Moreover, compared to mice, transplanted human hepatocytes displayed lagged xeno‐repopulation kinetics in rats, which could be partially attributed to larger liver size.^[^ ^22^ ^]^ Together, all these differences implicated the potential difficulty of xeno‐repopulation of PHHs in *Fah^–/–^* rats. It is apparent that strategies used for mice ought to be optimized substantially for the development of humanized liver in rats.

Here, we generated severely immunodeficient *Fah^–/–^Rag2^–/–^IL2rg^–/–^* (FRG) rats for PHH xenotransplantation. Indeed, all regular methods used in *Fah* ^–/–^ or FRG mice to induce liver injury could not be applied during liver xeno‐repopulation of human hepatocytes in FRG rats because of their extremely high mortality after NTBC withdrawal. A survival‐assured liver injury preconditioning (SALIC) protocol was developed for achieving a robust xeno‐repopulation of human hepatocytes in FRG rats. The rat model with humanized liver was characterized for its potential advantages in pharmacological studies and for the expansion of functional human hepatocytes in large quantities. In addition, the SALIC protocol for inducing chronic liver injury with assured survival of recipients might shed light on the transplantation of human hepatocytes in other large‐sized species.

## 2 Results

### 2.1 *Fah^–/–^Rag2^–/–^IL2rg^–/−^* Rats Develop Acute Liver Failure after NTBC Withdrawal

We generated severely immunodeficient *Rag2* ^–/–^ *IL2rg* ^–/‐^ (RG) rats using the CRISPR/Cas9 system (Figures S1 and S2A, Supporting Information). Abnormal lymphoid development of RG rats was validated by histological analyses (Figure S2B, Supporting Information). Importantly, CD3^+^CD45RA^–^ T cells, CD3^–^CD45RA^+^ B cells, and CD3^–^CD161a^+^ NK cells were depleted in the spleen as shown by flow cytometry analyses (Figure S2C, Supporting Information). In addition, IgG, IgM, and IgA were undetectable in RG rats (Figure S2D–F, Supporting Information).

**Figure 1:**
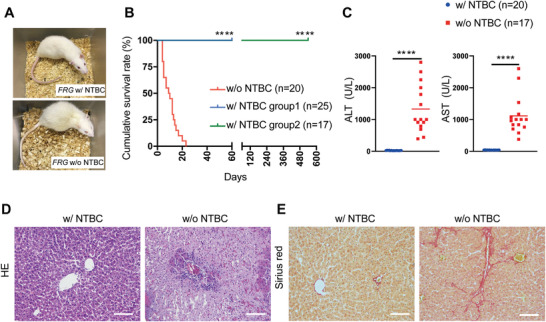
*Fah^–/–^Rag2^–/–^IL2rg^–/–^* rats developed acute liver failure after NTBC withdrawal. A) Images of FRG rats living with NTBC treatment or without NTBC treatment for 2 weeks. B) Kaplan–Meier survival curve of FRG rats with and without NTBC treatment. 42 FRG rats with NTBC treatment were divided into two groups: group 1 (*n* = 25, the blue line) was used for short‐term observation (60 days) and group 2 (*n* = 17, the green line) was for long‐term observation (540 days). ****p < 0.0001, log‐rank test. C) Serum ALT and AST levels in FRG rats with NTBC treatment and moribund FRG rats within 3 weeks after NTBC withdrawal. D,E) Representative images of D) HE and E) Sirius red staining of livers from normal FRG rats with NTBC treatment and moribund rats within 3 weeks after NTBC withdrawal. Scale bar: 100 µm. The data are shown as mean ± SD. ****p < 0.0001, Student's *t* test.

RG rats were crossed with *Fah* ^–/–^ rats^[^ ^19^ ^]^ to breed into FRG rats. During a continuous process to feed NTBC in drinking water, FRG rats could survive for more than 1.5 years (**Figure** **1**A,B), providing a sufficient time span for studying liver xeno‐repopulation and the following characterizations. After NTBC withdrawal, FRG rats developed acute liver failure, evidenced by elevated serum levels for ALT and AST as well as local massive necrosis in their liver (Figure 1C,D). Different from *Fah* ^–/–^ rats that developed liver cirrhosis after NTBC withdrawal,^[^ ^19^ ^]^ FRG rats only showed moderate liver fibrosis (Figure 1E). Notably, all FRG rats died within 4 weeks after NTBC withdrawal, and the median survival time was 9.5 days (Figure 1B). FRG rats died much faster than FRG mice, whose death usually occurred 4–8 weeks after NTBC withdrawal.^[^ ^2^ ^]^ These findings suggested that NTBC withdrawal‐induced liver injury was extremely harmful to FRG rats, which may hamper the manipulation process for hepatocyte transplantation and liver repopulation.

### 2.2 Characterization of the Xenotransplantation of Mouse Hepatocytes into the Livers of FRG Rats

FRG rats were characterized for the repopulation of transplanted hepatocytes. We first determined the number of hepatocytes required for liver repopulation. Syngeneic wild‐type rat hepatocytes ranging from 0.5 to 10 million were intraportally transplanted into FRG rats at 6–10 weeks of age, and NTBC feeding was removed after transplantation. FRG rats receiving 0.5 million hepatocytes died within 1 month (Figure S3A, Supporting Information). When the number of transplanted hepatocytes increased to 2.5 million, accounting for ≈1/500 of total rat hepatocytes, 54% of rat recipients survived liver failure‐induced death and showed 78 ± 11% of liver repopulation 2 months after transplantation (Figure S3A–C, Supporting Information). Remarkably, a further increase in donor hepatocytes (5 or 10 million) did not significantly improve survival rate (Figure S3A, Supporting Information), although repopulation rates were increased slightly (Figure S3B,C, Supporting Information). Given the high repopulation rates and the comparable survival rates, transplantation with 2.5 million donor hepatocytes was selected in our following analyses.

**Figure 2:**
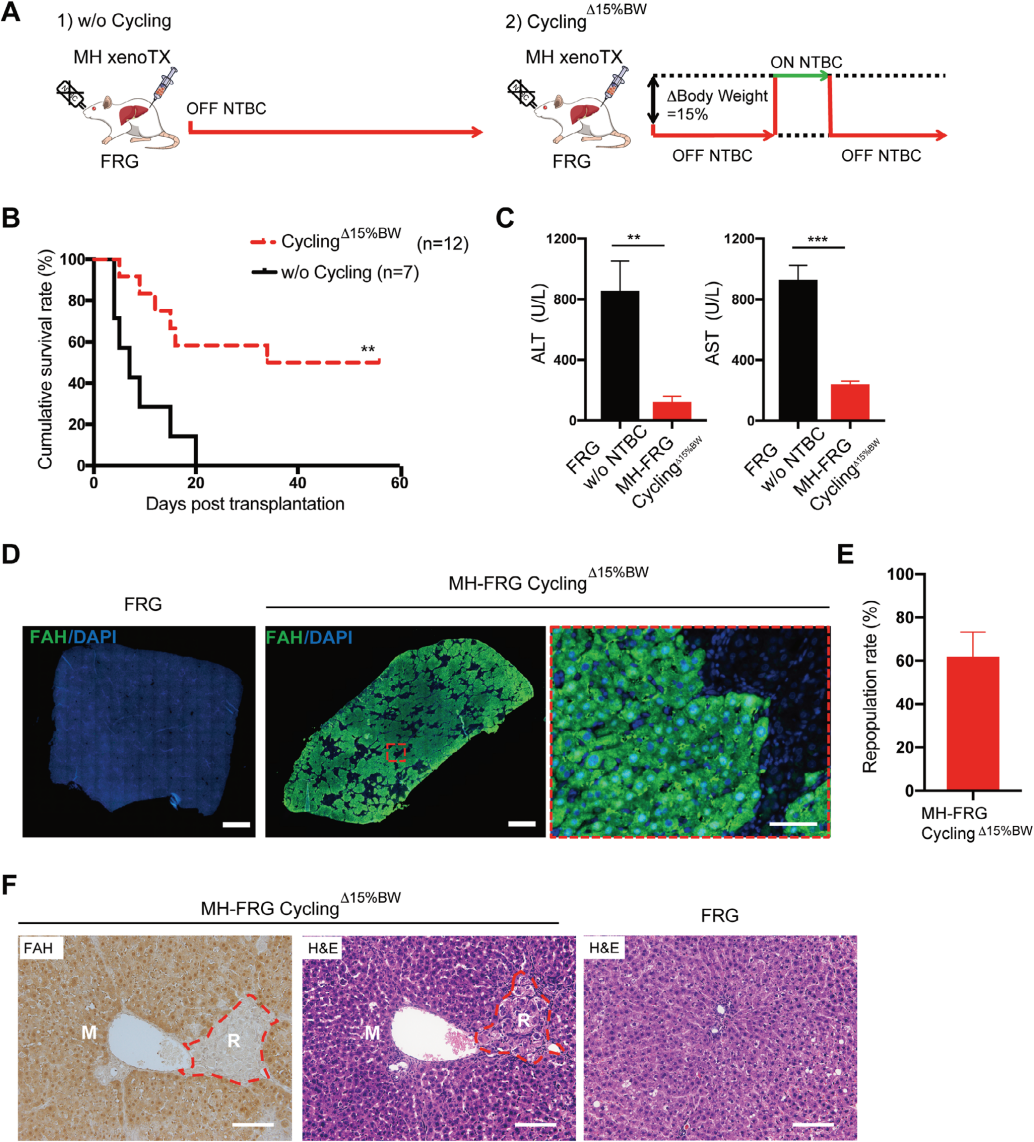
FRG rats as a xeno‐repopulation model for mouse hepatocytes. A) Schematic outline of mouse hepatocyte (MH) transplantation into the livers of FRG rats. After MH transplantation, NTBC was either permanently withdrawn from drinking water (w/o cycling) or repeatedly supplied dependent on the change in body weight. When over 15% body weight was lost, NTBC was transiently put on for 4 days (cycling^Δ15% BW^). B) Kaplan–Meier survival curve of MH‐transplanted FRG rats with or without NTBC cycling (Cycling^Δ15% BW^). C) Serum ALT and AST levels in moribund FRG rats within 2 weeks after NTBC withdrawal (FRG w/o NTBC) and in MH‐transplanted FRG rats with NTBC cycling for 8 weeks (MH‐FRG cycling^Δ15% BW^), *n* = 4. D) The repopulation of MHs was analyzed by FAH staining in the FRG rat livers with NTBC cycling^Δ15% BW^ 8 weeks after transplantation, and E) the repopulation rate (*n* = 4) was determined by FAH staining. Scale bar, 1000 and 50 µm (zoom in). F) FAH and HE staining of serial liver sections from MH‐FRG rats 8 weeks after transplantation. M, mouse; R, rat. Scale bar, 100 µm. The data are shown as mean ± SD. **p < 0.01, ***p < 0.001, log‐rank test for (B) and Student's *t* test for (C).

It was reported that rat hepatocytes showed almost identical liver repopulation kinetics as mouse hepatocytes (MHs) in FRG recipient mice.^[^ ^23^ ^]^ To understand xenotransplantation in FRG rats, we first transplanted MHs into the liver. All FRG rats receiving 2.5 million MHs died within 3 weeks after NTBC withdrawal (**Figure** **2**A,B). Small clusters of FAH^+^ donor hepatocytes were observed in livers of FRG recipient rats, proving the engraftment and repopulation of MHs but at relatively low levels (Figure S3D, Supporting Information). To improve xeno‐repopulation efficiency, we performed NTBC cycling, a protocol previously used to maintain survivals of *Fah^–/–^* mice during liver repopulation.^[^ ^2^, ^6^, ^24^ ^]^ Briefly, NTBC treatment was restarted when the recipient body weight was reduced by 15%, a critical point at which most animals could not survive further body weight loss (NTBC cycling^Δ15%BW^) (Figure 2A). Under NTBC cycling^Δ15%BW^, 6 of 12 FRG rat recipients successfully survived to 8 weeks after transplantation (Figure 2B). Levels of serum ALT and AST were significantly reduced in survived FRG rat recipients (Figure 2C). As determined by immunofluorescent assay of FAH and whole slide imaging (see the Experimental Section), MHs repopulated 62 ± 11% of recipient livers (Figure 2D,E). Morphologically, repopulated MHs were similar with rat hepatocytes in recipient livers (Figure 2F). Together, these results indicated that robust liver xeno‐repopulation from MHs could be successfully obtained in FRG rats. Overall, optimal control of the survival rate is the major issue for transplantation in FRG rats.

### 2.3 Establishment of Optimal Survival‐Assured Liver Injury Preconditioning for the Expansion of Human Hepatocytes

**Figure 3:**
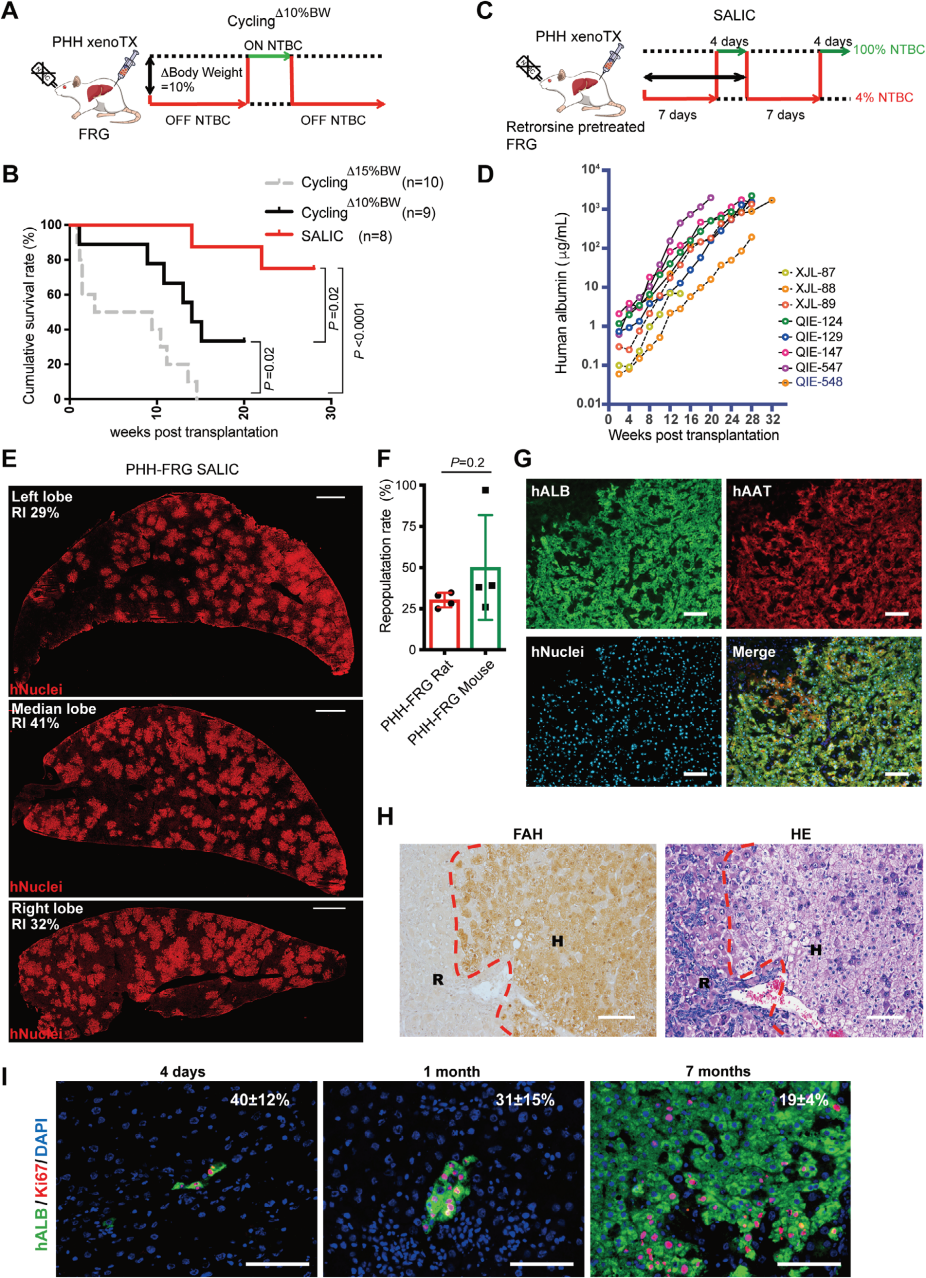
PHHs robustly repopulate the livers of FRG rats. A) Schematic outline of PHH transplantation with NTBC cycling^Δ10% BW^. B) Kaplan–Meier survival curve of PHH‐transplanted FRG rats under NTBC cycling^Δ10% BW^, NTBC cycling^Δ15% BW^, and SALIC. The survival data of NTBC cycling^Δ15% BW^ is from the same experiment in Figure S4A in the Supporting Information. C) Schematic outline of PHH transplantation with SALIC. FRG rats were pretreated with retrorsine 2 weeks before transplantation. The rats were repeatedly administrated with 4% NTBC (0.2 mg L^−1^) for 7 days to induce liver injury and subsequently 100% NTBC (5 mg L^−1^) for 4 days to allow recovery. D) The dynamic change of human ALB secretion in PHH (lot: XJL and QIE)‐transplanted FRG rats in 28 weeks under SALIC. E) Repopulation of PHHs was estimated by human nuclei antigen (hNuclei) staining in the FRG rat livers 7 months after transplantation. Representative images of the left, median, and right liver lobes from the same humanized rat were shown. Replacement index (RI) is the ratio of the area of repopulated human hepatocytes to the area of the host liver lobule. Scale bar, 2 mm. F) The repopulation rates of PHHs from the same donor were determined in FRG rats (*n* = 4) and mice (*n* = 4) by hNuclei or FAH staining 7 months after transplantation. The representative images of FAH staining were shown in Figure S6E in the Supporting Information. The data are shown as mean ± SD. *p* = 0.2, Student's *t* test. G) The expression of mature hepatic markers of repopulated PHHs was analyzed by co‐staining of human ALB, AAT, and hNuclei. Scale bar, 100 µm. H) FAH and HE staining of serial liver sections from PHH‐FRG rats 7 months after transplantation. Regions that contain FAH‐positive and negative hepatocytes were defined as human (H) and rat (R) areas, respectively; the boundary is indicated by a dashed line. Scale bar, 100 µm. I) The proliferation of human hepatocytes in FRG rat livers was determined by co‐staining of Ki67 and human ALB (hALB), *n* = 3. The ratio of Ki67^+^ hALB^+^ hepatocytes to total hALB^+^ hepatocytes was shown. Scale bar, 100 µm. The data are shown as mean ± SD.

Human hepatocyte transplantation was performed in FRG rats based on the conditioning protocol obtained from MH transplantation. After 2.5 million cryopreserved PHHs were intraportally transplanted, FRG rat recipients underwent NTBC cycling^Δ15%BW^. Five of 10 FRG rat recipients died within the first month, and all other FRG rat recipients died within 4 months (Figure S4A, Supporting Information). To improve recipient survival, the NTBC cycling protocol was optimized when the body weight of FRG rat recipients was reduced by 10% (NTBC cycling^Δ10%BW^, **Figure** **3**A) instead of 15%. Under NTBC cycling^Δ10%BW^, eight of nine FRG rat recipients transplanted with PHH successfully survived after 1 month. Three of nine FRG rat recipients further survived after 5 months (cycling^Δ10%BW^ vs cycling^Δ15%BW^, *p* = 0.02) (Figure 3B) and showed a significant improvement in liver function (Figure S4B, Supporting Information). Human albumin (hALB) was secreted substantially to 0.8 ± 0.4 mg mL^−1^ 5 months after PHH transplantation (Figure S4C, Supporting Information). Levels of PHH xeno‐repopulation were 15 ± 11% as determined by the FAH immunoassay (Figure S4D, Supporting Information), which was far below the expected PHH repopulation efficiency (30%) for functional assays.^[^ ^5^ ^]^ Moreover, the high death rate (6/9) made it difficult to apply this protocol. Obviously, the NTBC cycling^Δ10%BW^ protocol should be further optimized to improve liver xeno‐repopulation efficiency in FRG rats.

Liver injury is essential for the repopulation of transplanted PHHs. However, it should be controlled at a proper level without killing FRG rat recipients before robust repopulation of transplanted PHHs. While analyzing moribund FRG rats under NTBC cycling^Δ10%BW^, we found that body weight loss did not change concomitantly with elevated ALT and AST levels, suggesting that body weight loss did not reliably reflect the severity of liver failure (Figure S4E, Supporting Information). We therefore decided to control chronic liver injury more precisely by manipulating NTBC concentrations and cycling intervals rather than monitoring body weight loss. Instead of full removal of NTBC, FRG rats were treated with reduced doses of NTBC to induce liver injury. The colony maintenance dose of NTBC (5 mg L^−1^) was taken as 100% NTBC. An NTBC treatment of 1% led to the mortality of FRG rats comparable to those with complete NTBC removal, whereas 10% NTBC treatment only caused mild liver injury (Figure S5A,B, Supporting Information). Notably, 4% NTBC (0.2 mg L^−1^) treatment induced chronic liver injury efficiently and extended the median survival time to 22 days (Figure S5A,B, Supporting Information), which almost doubled that of FRG rats with complete NTBC removal and provided an extended time window to manipulate NTBC cycling.

To maximize liver injury while keeping FRG rats alive, we determined the optimal duration of low‐dose NTBC treatment in the NTBC cycling protocol (Figure S5C, Supporting Information). NTBC cycling with 4% NTBC for 9 days to induce liver injury followed by 100% NTBC for 4 days for recovery resulted in a mortality of 50% at around 40 days, which was too high for PHH transplantation (Figure S5D, Supporting Information). When FRG rats treated with 4% NTBC for 5 or 7 days during NTBC cycling, survival rates were increased to 2 months (Figure S5D, Supporting Information). Moreover, when livers from these rats were analyzed, 4% NTBC treatment for 7 days induced higher liver injury as manifested by increased fibrosis (Figure S5E, Supporting Information). NTBC cycling was finally determined using 4% NTBC for 7 days followed by 100% NTBC for 4 days (NTBC cycling with fixed treatment, NTBC cycling^FT^).

Notably, around 3% of hepatocytes were Ki67 positive in FRG rats after treatment of NTBC cycling^FT^, suggesting it created a regenerative microenvironment (Figure S5F, Supporting Information). To exploit the regenerative microenvironment only for transplanted hepatocytes, we blocked the proliferation of endogenous hepatocytes with retrorsine treatment. Retrorsine pretreatment before NTBC cycling^FT^ remarkably inhibited the proliferation of endogenous hepatocytes (Figure S5F, Supporting Information). Together, the combination of retrorsine pretreatment and NTBC cycling^FT^ was established as the SALIC protocol for liver xeno‐repopulation of human hepatocytes.

### 2.4 Achievement of Robust Liver Xeno‐Repopulation of Human Hepatocytes in FRG Rats

PHHs were transplanted into FRG rats following the SALIC protocol (Figure 3C). Remarkably, seven of eight FRG rat recipients survived after 5 months (Figure 3B). Survival rate significantly increased when the SALIC protocol was compared to NTBC cycling^Δ15%BW^ and NTBC cycling^Δ10%BW^ (*p* < 0.0001 and *p* = 0.02, respectively; Figure 3B). Meanwhile, there was no significant difference in survival curves between rats transplanted with two different PHHs (*p* = 0.61, Figure S6A, Supporting Information). Importantly, the level of hALB secretion continuously increased to an average of 1.7 ± 0.3 mg mL^−1^ in five of six survived FRG rat recipients after 7 months (Figure 3D). One recipient showed that hALB levels plateaued at ≈200 *μ*g mL^−1^, probably because of low engraftment of transplanted PHH since the beginning (Figure 3D). The five remaining rats with hALB levels >1 mg mL^−1^ were further characterized.

Macroscopic analyses indicated that humanized rat livers were normal in shape and showed similar liver/body weight ratios when compared with nontransplanted livers (Figure S6B, Supporting Information). Immunofluorescent staining of human‐specific nuclei antigen (Figure S6C, Supporting Information) demonstrated a consistent level of liver xeno‐repopulation from human cells as high as 31 ± 4% (Figure 3E,F). Co‐staining for human FAH, ALB, and AAT further confirmed the human origin of repopulated hepatocytes (Figure 3G and Figure S6D, Supporting Information). The immunofluorescent staining of hNuclei or FAH suggested the repopulation rate in FRG rats was comparable with that in FRG mice transplanted with the same donor PHHs (31 ± 4% vs 50 ± 31%; *p* = 0.2) (Figure 3F and Figure S6E, Supporting Information). Because a rat liver has roughly 1 billion hepatocytes, if engraftment efficiency of transplanted PHHs was estimated at 10%,^[^ ^25^ ^]^ it was calculated that the engrafted PHHs expanded around 1200 times up to 300 million in vivo. In addition, there was no significant difference in repopulation rates between rats transplanted with two different PHHs (15 ± 14% vs 32 ± 3%; *p* = 0.12). Overall, these data suggested the reproducibility of the SALIC regimen.

FAH^+^ human hepatocytes were completely integrated in rat liver parenchyma without disturbing the normal liver structure (Figure 3H). The repopulated human hepatocytes could be distinguished from rat hepatocytes by showing pale cytoplasm staining of eosin and increased accumulation of glycogen (Figure 3H and Figure S6F, Supporting Information). Remarkably, there were around 10% hALB^+^Ki67^+^ human hepatocytes at 7 months after transplantation, holding the potential to achieve even higher liver humanization (Figure 3I). It was noteworthy that no tumors originating from either human or rat hepatocytes were detected in humanized livers as determined by pathological analysis and AFP staining (Figure 3H and Figure S6G, Supporting Information).

Together, these findings indicated that a notable level of liver humanization was established in FRG rat recipients by SALIC. In following analyses, human‐specific liver metabolism, in terms of transcriptome, zonation, and drug metabolism, were characterized in FRG rats of humanized liver.

### 2.5 Humanized Rat Liver Maintains the Transcriptome Features of Human Liver

**Figure 4:**
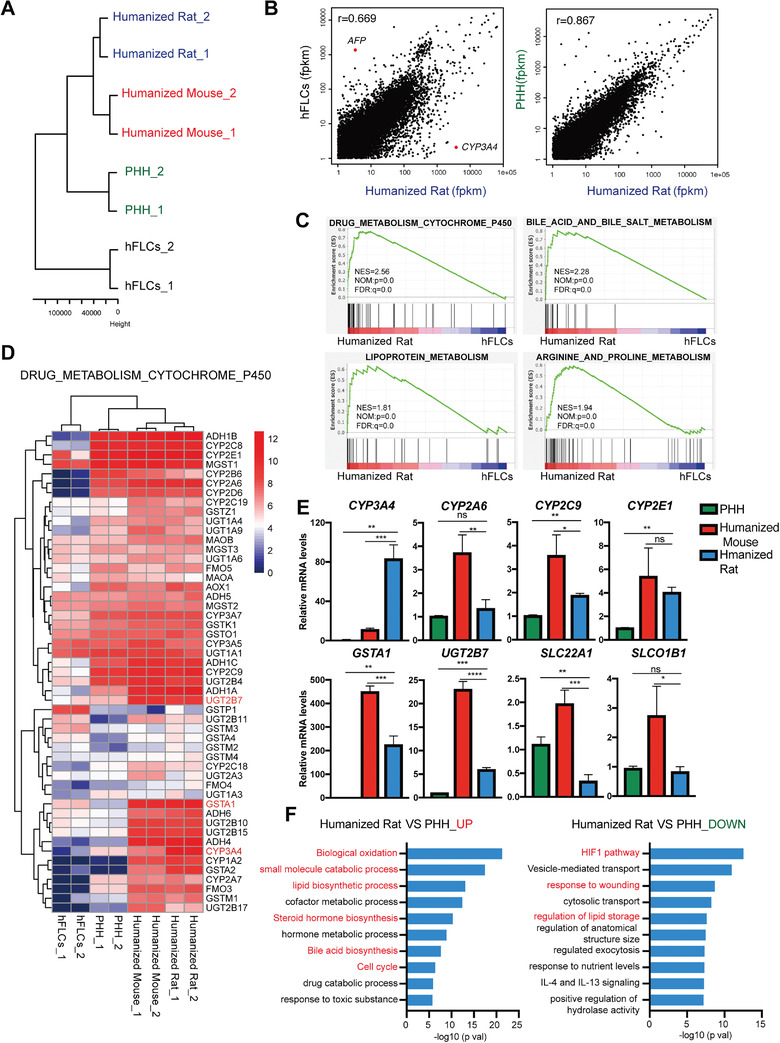
The gene expression profile of humanized livers in FRG rats resembles that of human livers. A) Unsupervised hierarchical clustering of RNA‐seq data reveals similarities between freshly thawed PHH and liver‐humanized rat livers (humanized rat). Human fetal liver cells (hFLCs) and liver‐humanized mouse liver (humanized mouse) were used as controls. PHHs from the same donor were used. B) Scatterplot of RNA sequencing of human hepatocyte transcriptomes from hFLCs, PHH, and liver‐humanized rat livers. C) Gene set enrichment analysis (GSEA) was performed to identify enriched pathways between liver‐humanized rat livers and hFLCs. D) Heat map and hierarchical clustering of expression of genes involved in drug metabolism pathways from RNA‐seq data were analyzed in hFLCs, PHH, liver‐humanized mouse, and rat livers. E) Comparison of gene expression of mature hepatic markers, including phase I, phase II enzymes, and transporter genes in PHHs, liver‐humanized mouse (*n* = 3), and rat (*n* = 3) livers. Human‐specific primers were used in qPCR. The data are shown as mean ± SD. ns *p* > 0.05, *p < 0.05, **p < 0.01, ***p < 0.001, Student's *t* test. F) Gene ontology analysis was performed to identify enriched pathways in liver‐humanized rat livers when compared to PHHs.

The xenogeneic microenvironment in the liver of rodent species may influence the gene expression of repopulated human hepatocytes.^[^ ^26^ ^]^ To evaluate whether repopulated human hepatocytes in FRG rats maintained the typical transcriptome of mature human hepatocytes, human‐specific RNA‐sequencing analysis was performed on liver homogenates from the humanized livers of FRG rats (see the Experimental Section). The humanized liver of FRG mouse^[^ ^24^ ^]^ generated from the same donor hepatocytes was also used as a control. Human fetal liver cells (hFLCs)^[^ ^27^ ^]^ were used as the control of immature hepatocytes. The expression profile analysis demonstrated humanized rat livers clustered closer to donor PHHs and humanized mouse livers than hFLCs (**Figure** **4**A). Pearson's correlation coefficient was further calculated to determine similarities between PHHs and humanized livers. Humanized rat livers showed a higher level of correlation to donor PHHs (*r* = 0.867) than to hFLCs (*r* = 0.669) (Figure 4B and Figure S7A, Supporting Information).

Humanized rat livers displayed significant enrichment in the expression of genes involved in hepatocyte functions by gene set enrichment analysis (GSEA; Figure 4C). Specifically, humanized rat livers maintained the gene expression of liver metabolism pathways (Figure 4D and Figure S7B,C, Supporting Information). RNA sequencing data of some metabolism genes of PHHs were further confirmed by quantitative polymerase chain reaction (qPCR) using human‐specific primers, including ALB; phase I enzymes CYP3A4, CYP2A6, CYP2C9, and CYP2E1; phase II enzymes UGT2B7 and GSTA1; and transporters SLC22A1 and SLCO1B1. These metabolism genes were expressed in humanized rat livers at levels comparable with those in PHHs (Figure 4E and Figure S7D, Supporting Information). By contrast, humanized rat livers did not show an expression of AFP, a marker of fetal liver cells, supporting the maintenance of a mature phenotype of PHHs after robust repopulation in FRG rats (Figure S7E, Supporting Information).

Intriguingly, whereas 86.2% of total genes were expressed similarly between PHH and humanized rat liver, 2209 genes showed differential expression (Figure S7F,G, Supporting Information). We explored the biological implications of the differentially expressed genes (DEGs) between humanized rat livers and PHHs using gene ontology analysis. The upregulated genes in humanized rat livers displayed enrichment in pathways related to the small molecule metabolism, lipid and steroid hormone biosynthesis, and cell cycle (Figure 4F). These upregulated metabolism pathways were also enriched in humanized mouse livers (Figure S7H, Supporting Information), suggesting the influence of xenogeneic microenvironment in vivo^[^ ^26^ ^]^ or reflecting the difference between repopulated PHH and cryopreserved PHH. The upregulation of cell cycle genes was consistent with the proliferative state of repopulated PHHs. On the other hand, the downregulated genes in humanized rat livers showed enrichment in pathways related to the hypoxia‐inducible factor (HIF) pathway and the regulation of lipid storage (Figure 4F). The downregulation of VEGF*α* and VEGF*β* expression in the HIF pathway might partially account for low vessel density in PHH‐repopulated clones (Figures S7G and S8, Supporting Information). The increase of lipid biosynthesis and dysregulation of lipid storage were in line with slightly enhanced lipid accumulation of repopulated PHHs as shown by hematoxylin and eosin (HE) staining (Figure 3H). Collectively, these results indicated that humanized rat livers with over 30% chimerism largely retained the expression of mature human hepatocyte genes.

### 2.6 Human‐Specific Metabolic Architecture Is Established in Humanized Rat Livers

**Figure 5:**
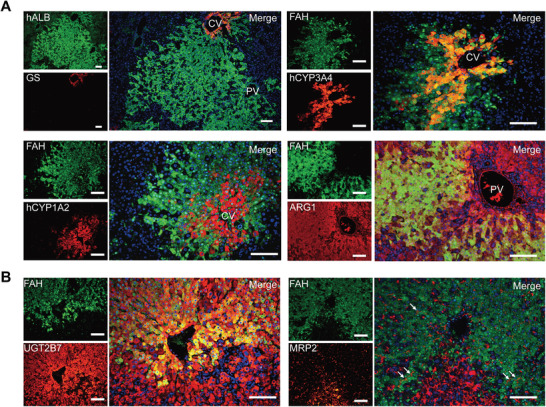
Liver zonation of metabolism in humanized rat liver. A) The liver zonation was determined by staining for human ALB, FAH, CYP3A4, CYP1A2, and ARG1. CV, central vein; PV, portal vein. Scale bar, 100 µm. B) The expression of phase II enzymes and transporters was representatively analyzed by staining for FAH, UGT2B7, and MRP2. Scale bar, 100 µm.

Lobule zonation is the fundamental architecture of the liver.^[^ ^28^ ^]^ Hepatocytes along the porto‐central axis of the liver lobule show remarkable heterogeneity with respect to metabolic functions. Under such circumstance, the key metabolic enzymes are preferentially expressed in periportal or pericentral hepatocytes.^[^ ^29^ ^]^ Previous studies revealed that human‐specific liver zonation was formed in humanized mouse livers with essential human liver metabolism.^[^ ^9^ ^]^ We investigated whether human‐specific metabolism was successfully established in livers of FRG rats, featuring proper human liver zonation. Immunofluorescent assay validated the pericenter‐specific expression of glutamine synthetase in repopulated human hepatocytes (**Figure** **5**A). In addition, expressions of pericenter‐specific phase I enzymes, CYP3A4 and CYP1A2, were only found in the repopulated human hepatocytes of pericentral regions by using human‐specific immunofluorescent assay (Figure 5A). By contrast, periportal‐specific ARG1 showed an expression pattern restrictive to periportal areas composed of repopulated human hepatocytes (Figure 5A). Furthermore, both ubiquitously expressed phase II enzyme UGT2B7 and transporter MRP2 were found in repopulated human hepatocytes along the liver lobule (Figure 5B). Such expression pattern was consistent with that in human liver lobules.

### 2.7 Human‐Specific Drug Metabolism Is Established in Humanized Rat Liver

**Figure 6:**
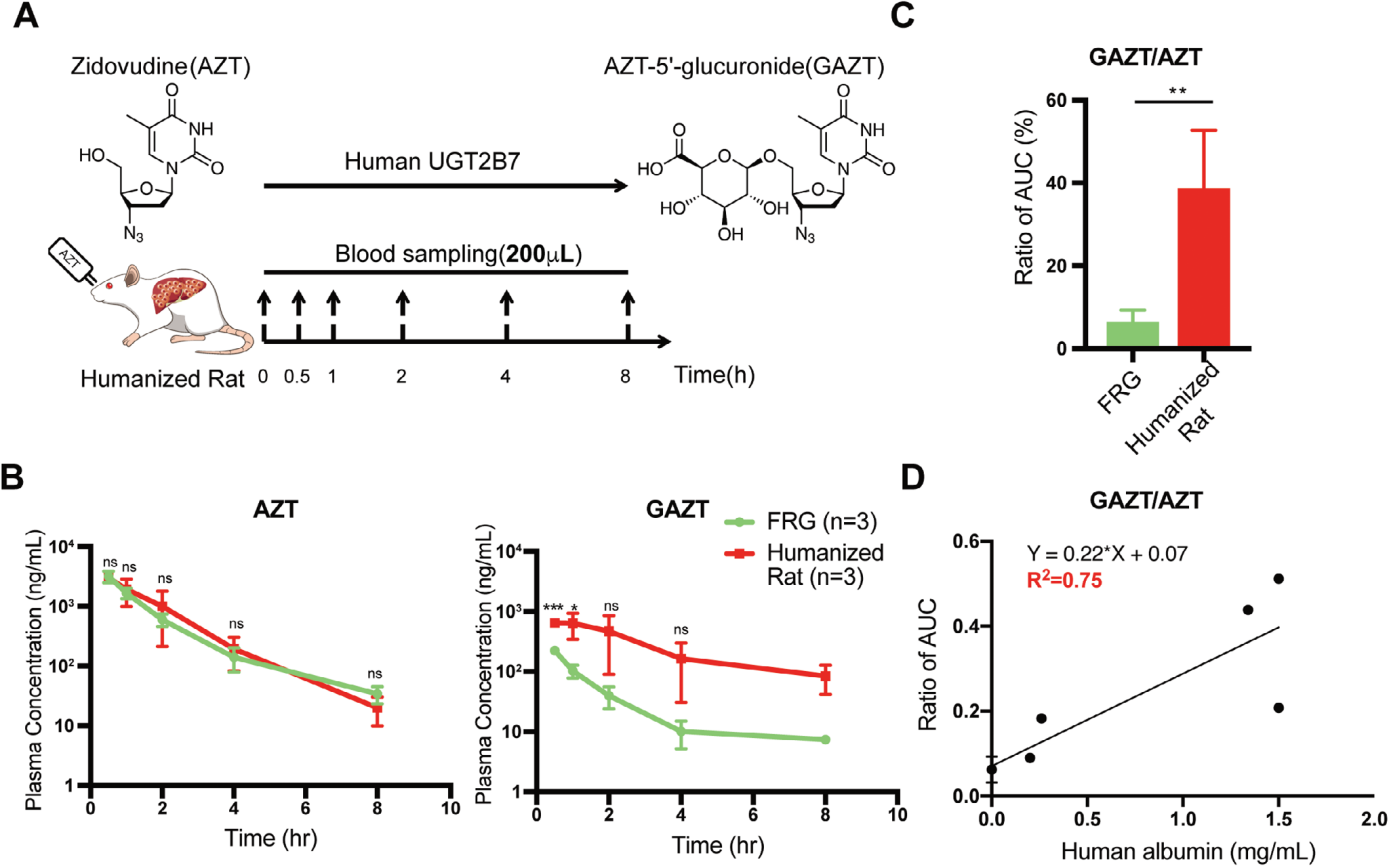
Human‐specific drug metabolism in liver‐humanized rats. A) Schematic outline of the analysis of human UGT2B7‐specific metabolism of zidovudine (AZT) in liver‐humanized FRG rats (humanized rat). The rats were orally administered with AZT (15 mg kg^−1^), and 200 uL of blood was collected at indicated time points. B) Time‐dependent change in the concentration of AZT and AZT‐5′‐glucuronide (GAZT) in FRG rats with or without liver humanization. C) Ratio of AUC for GAZT/AZT in FRG rats with (*n* = 3) or without (*n* = 4) liver humanization. Liver‐humanized rats with hALB secretion levels around 1.5 mg mL^−1^ were used. D) The correlation between the ratios of AUC (GAZT/AZT) and human ALB secretion levels in the liver humanized rats. The data are shown as mean ± SD. ns *p* > 0.05, *p < 0.05, **p < 0.01, ***p < 0.001, Student's *t* test.

Human‐specific drug metabolism was one of the most expected features of humanized livers of FRG rats. Based on previous studies,^[^ ^2^, ^5^ ^]^ liver‐humanized FRG rats with a serum level of hALB >1 mg mL^−1^ were used to prove this specific feature. Characterization of the metabolic activity of human‐specific phase II enzyme UGT2B7 was chosen because it involves the metabolisms for about one‐third of all drugs presently used in the clinic.^[^ ^30^ ^]^ Zidovudine (AZT), the first approved drug for HIV treatment,^[^ ^31^ ^]^ was specifically used as a probe substrate for UGT2B7 activity in glucuronidation. In human liver, AZT is converted by UGT2B7 to AZT‐5′‐glucuronide (GAZT)^[^ ^32^ ^]^ (**Figure** **6**A). By contrast, less than 10% of the administered dose of AZT was converted into GAZT in rat livers. To monitor the human‐specific metabolism of orally administrated AZT, 200 µL of blood samples were collected for six time points (1.2 mL in total) at 0, 0.5, 1, 2, 4, and 8 h (Figure 6A), a volume not possible for mice. The area under the plasma concentration–time curve (AUC) of AZT and GAZT was measured (Figure 6B). The similarity between AUC_0‐_ *_t_* and AUC_0‐∞_ confirmed the sampling time points were sufficient to analyze pharmacokinetics (Table S1, Supporting Information). The peak concentration (*C*_max_) of AZT showed no significant difference between liver‐humanized rats and FRG rats (3060.8 ± 102.0 and 3196.4 ± 694.5 ng mL^−1^, respectively; *p* = 0.7547) (Figure 6B and Table S1, Supporting Information). Notably, liver‐humanized rats showed significantly increased *C*_max_ of GAZT compared with the control group (725.1 ± 166.6 and 222.3 ± 32.9 ng mL^−1^, respectively; *p* = 0.0068) (Figure 6B and Table S1, Supporting Information). Moreover, the AUC ratios of GAZT and AZT (GAZT/AZT) were 39 ± 16% and 6 ± 3% in humanized and control rats, respectively, demonstrating human‐specific UGT2B7 activity in liver‐humanized FRG rats (Figure 6C). Additionally, only rats with hALB levels > 1 mg mL^−1^ showed significantly increased UGT2B7 activity, further suggesting the metabolic activity of UGT2B7 was highly correlated to chimerism (Figure 6D). Together, these data suggested that liver‐humanized rats could be used as a model to reflect human‐specific drug metabolism.

## 3 Discussion

Liver‐humanized animals have become the state‐of‐the‐art model systems to mimic both physiological and pathological features of human livers.^[^ ^3^, ^33^ ^]^ Liver humanization has been studied in several species, including mice, rats, and pigs, but it is only successfully accomplished in mice. Here, we reported the establishment of humanized liver in FRG rats, which will be favored in pharmacological studies.

More than four mouse models were successfully generated with efficient liver‐humanization through either hepatotoxic transgene expression or gene knockout.^[^ ^3^, ^33^ ^]^ Among them, *Fah* knockout showed some extraordinary advantages, such as controllable liver injury and easiness in genetic manipulation, and thus became the main strategy for liver humanization in other animals.^[^ ^34^ ^]^ Intriguingly, *Fah* ^–/–^ mice showed significant discrepancies in pathology when compared to Fah‐deficient animals of other species.^[^ ^19^, ^35^ ^]^ Moreover, previous findings suggested delayed repopulation kinetics of transplanted human hepatocytes in rats.^[^ ^22^ ^]^ In this study, we found that FRG rats were sensitive to NTBC withdrawal‐induced liver injury and died faster compared with FRG mice. These features of FRG rats prevented a direct application of the precondition protocol used for FRG mice. We thus developed SALIC, which combined both retrorsine pretreatment and optimized NTBC cycling^FT^, to assure survival while maintaining the regenerative microenvironment induced by chronic liver injury. Under such optimized precondition, the engrafted human hepatocytes maintained a continuous proliferation up to > 7 months after xenotransplantation, the underlying mechanism of which is not yet understood. Notably, SALIC improved both the survival of FRG rats and liver xeno‐repopulation rate without detectable liver tumorigenesis. By contrast, long‐term (> 6 months) NTBC cycling would induce liver cancers in FRG mice.^[^ ^36^ ^]^ It is possible that the treatment of low‐dose NTBC induced low levels of liver injury and limited the accumulations of mutagenic molecules.^[^ ^37^ ^]^ It is conceivable that transient retrorsine pretreatment might also contribute to undetectable tumorigenesis.^[^ ^38^ ^]^

Repopulation kinetics of transplanted hepatocytes is largely influenced by liver size. The precondition learned from rats might shed light on hepatocyte transplantation in species of large sizes. Humanized livers generated in pigs were proposed as alternative organs for clinical liver transplantation^[^ ^39^ ^]^ given the similarity between pig livers and human livers in size and anatomy. Fah‐deficient rat and pig both developed acute liver failure after NTBC withdrawal^[^ ^40^ ^]^ and were sensitive to retrorsine‐mediated inhibition of hepatocyte proliferation.^[^ ^41^ ^]^ It may be worth testing whether the principle of SALIC could be applied to liver humanization in *Fah* ^–/–^ pigs. Furthermore, it would be interesting to propose SALIC as a useful reference for hepatocyte transplantation in human patients.

Predicting both human‐specific drug metabolite profiles and pharmacokinetics in vivo is important for estimating drug efficacy and toxicity. For drugs such as AZT, only primates were chosen for toxicity studies because they had a metabolic profile (i.e., GAZT formation) similar to that of humans.^[^ ^32^ ^]^ Notably, liver‐humanized rats display human‐specific drug metabolism in terms of metabolite profiles and pharmacokinetics, which can be an alternative model to primates for such toxicity analysis. Despite the significantly high GAZT formation in liver‐humanized rats, the pharmacokinetics of AZT was similar between liver‐humanized rats and FRG control rats. It was possible that AZT was eliminated by multiple mechanisms, e.g., the important contribution of renal clearance. Technically, it demands sequential blood sampling from one tested animal to complete the pharmacokinetic study.^[^ ^42^ ^]^ We showed that one liver‐humanized rat could provide at least 1.2 mL of blood sample without adverse effects on physiology. It is impossible to collect such volume of blood samples from mice, as it approximately equals the whole blood volume for one mouse. It is apparent that liver‐humanized rats have unique advantages in testing drugs requiring sequential blood samplings.

Expanding PHHs and maintaining their mature phenotype at the same time remains challenging in vitro.^[^ ^19^, ^24^, ^43^ ^]^ In vivo expansion via humanized liver in animals is an alternative solution. We showed that repopulated human hepatocytes in FRG rats maintained a mature gene expression profile, providing a new system to expand human hepatocytes. Rats are ten times larger than mice, and one rat is capable of producing at least 1 billion human hepatocytes if fully repopulated. It would be possible to isolate large quantities of fully functional human hepatocytes from FRG rats to meet most applications, including high‐throughput drug screening and application in liver assist devices.^[^ ^3^ ^]^ In this study, an expansion of at least 1200 times had been achieved in FRG rats based on an initial engraftment of 0.25 million human hepatocytes (engraftment efficiency estimated at 10%) and a final harvest of about 300 million after 30% repopulation of rat livers. By contrast, an ≈150‐fold expansion of human hepatocytes was generally achieved in FRG mice.^[^ ^5^ ^]^

Despite their advantages, it took as long as 7 months to achieve 30% human chimerism in FRG rat livers, which apparently required further improvements in the efficiency of liver humanization. It was previously reported that the transplantation of an increased number of human hepatocytes reproducibly facilitated high human chimerism.^[^ ^6^ ^]^ In addition, it was reported that 70–80% of transplanted donor hepatocytes were cleared by the resident macrophage in liver.^[^ ^44^ ^]^ Compared with FRG mice on the C57BL/6J strain, liver humanization was established faster in those on the NOD strain,^[^ ^8^ ^]^ in which the signal regulatory protein alpha (Sirp*α*) binds to CD47 in human hepatocytes and thus decreased the phagocytosis of human hepatocytes by mouse macrophages via CD47‐Sirp*α* interaction (the so‐called “don't eat me” signal).^[^ ^44^ ^]^ The enhanced level of liver humanization may be also achieved in FRG rats with human Sirp*α* knock‐in. With these future improvements, humanized livers in FRG rats would serve as a model for studies on pharmacology and liver diseases and as an efficient tool for in vivo expansion of human hepatocytes.

## 4 Experimental Section

### Generation of *Fah^–/–^Rag2^–/–^IL2rg^–/–^* Rats

The Rag2 and IL2rg double mutant rats were generated by microinjection of sgRNA and Cas9 mRNA into wild‐type (WT) SD rat zygotes. Following crossing with *Fah* ^–/–^ rats, *Fah^–/–^Rag2^–/–^IL2rg^–/‐^* (FRG) rats were generated and fed with drinking water containing 5 mg L^−1^ NTBC (synthesized by Capot Chemical, China). All animal experiments were performed according to protocols approved by the animal care and use committee at the Shanghai Institute of Biochemistry and Cell Biology and the University of Tsukuba.

### Xenotransplantation of Human Hepatocytes in FRG Rats

The cryopreserved PHHs from four individuals (Lot: JFC, TVR, QIE, and XJL) were purchased from Celsis In Vitro Technologies (Baltimore, MD). In the pilot experiments, 6 days before cell transplantation, NTBC concentration in drinking water for FRG rats was first reduced to 2.5 mg L^−1^ for 3 days and was then totally withdrawn for another 3 days. Then, 2.5 × 10^6^ PHHs in 500 µL phosphate‐buffered saline (PBS) were transplanted through the portal vein into the liver of FRG rats. NTBC was transiently put on for 4 days when rats lost over 10% of their body weights (NTBC cycling^Δ10%BW^). Rats were sacrificed 5 months later. In the SALIC protocol, 30 mg kg^−1^ retrorsine (sigma) was intraperitoneally injected to FRG rats 2 weeks before transplantation. NTBC cycling with fixed treatment (NTBC cycling^FT^) was carried out 1 week before transplantation and lasted until the end of the experiment. Briefly, the rats were repeatedly administrated with 0.2 mg L^−1^ NTBC for 7 days to induce injury followed by 5 mg L^−1^ NTBC for 4 days to allow recovery. Rats were sacrificed 7 months later.

### Histology, Immunohistochemistry, and Immunofluorescence

These were performed according to the standard procedures as previously described.^[^ ^24^ ^]^ See the Supporting Information for details.

### RNA Sequencing Data Process

All sequencing reads from RNA‐seq were mapped to the human reference genome (hg38) using hisat2‐2.10. Thus, the transcripts derived from the repopulated human hepatocytes in humanized liver were specifically analyzed. Fragments per kilobase of exon per million fragments mapped (FPKM) values were calculated by Cufflinks v.2.2.1 using default parameters for gene expression levels. htseq‐count was used to count reads on genes. Differential expression analysis was performed using DESeq2 (R package). Genes were considered differentially expressed if FPKM > 1 in all sample and fold changed ≥ 2.5, padj ≤ 0.05. RNA‐seq data of hFLCs, PHH, and humanized mouse liver were adapted from GEO datasets (GEO: GSE112330 and GSE112866).

### Pathway Enrichment Analysis

GSEA was used for the pathway enrichment of DEGs. For the list of DEGs, the online MSigDB tool was used (http://software.broadinstitute.org/gsea/msigdb/index.jsp). GSEA v2 desktop software was also used to identify the significantly enriched pathways from the RNA‐seq data. Gene ontology analysis was performed using clusterProfiler v3.14.3 (R package).

### UGT2B7 Metabolism Assay

FRG rats with or without liver humanization were orally administered with AZT (15 mg kg^−1^, WAKO). 200 µL of blood was collected at 0, 0.5, 1, 2, 4, and 8 h. 30 µL plasma was diluted with 30 µL PBS for measurement of substrate and metabolite by liquid chromatography with tandem mass spectrometry (Agilent 1200 HPLC and ABI 4000 mass‐spectrometer). Substrate (AZT, Sigma) and metabolite (AZT‐5′‐Glucuronide, Toronto Research Chemicals) used for standard curves were commercially purchased.

### Statistical Analysis

The number of biological and technical replicates and animals are indicated in figure legends and text. All data were presented as mean ± SD. For most statistical evaluations, an unpaired Student's *t* test was applied for calculating statistical probability in this study. *p*‐Values were calculated by two‐tailed test. Only for survival analyses, the Mantel–Cox log‐rank test was applied. Statistical calculation was performed using GraphPad Prism 5 (GraphPad). Repopulation efficiency was analyzed using Keyence BZ‐X710 microscope and BZ‐X Analyzer software (Keyence). Briefly, the left, median, and right lobes from transplanted rats were harvested and stained using FAH or hNuclei antibody to detect repopulated donor hepatocytes. Whole slide imaging was further conducted to minimize the potential bias caused by high‐magnification views. The FAH^+^ or hNuclei^+^ areas were measured using Image J. Repopulation rate was calculated as: FAH^+^ or hNuclei^+^ area/total liver lobe area scanned × 100%.

### Data Resources

The accession number for the RNA‐seq data reported in this paper is GEO: GSE162862 (www.ncbi.nlm.nih.gov/geo/query/acc.cgi?acc=GSE162862).

## Conflict of Interest

The authors declare no conflict of interest.

## Author Contributions

L.Z., J.‐Y.G., and Y.‐W.Z. contributed equally to this work. L.H. and Y.‐W.Z. conceived and supervised the study. L.Z. and J.‐Y.G. designed and performed most of the experiments; Z.S. and C.W. assisted in the characterization of humanized rats; B.W., L.Z., and X.M. analyzed the RNA‐seq data; Z.P. and G.P. performed the analysis of UGT2B7 activity in vivo; D.L. and Y.S. generated *Fah^–/–^Rag2^–/–^IL2rg^–/–^* rats using CRISPR/Cas9; J.‐Y.G., Y.‐W.Z., K.F., and M.F. established NTBC cycling^FT^; N.O., T.O., J.F., L.H., and Y.‐W.Z. supplied experimental materials and resources; and L.Z., J.‐Y.G., Y.‐W.Z., and L.H. analyzed the data and wrote the manuscript.

## Supporting information

Supporting Information

## Data Availability

The data that support the findings of this study are openly available in GEO: GSE162862 at www.ncbi.nlm.nih.gov/geo/query/acc.cgi?acc=GSE162862.
