# Supplementary material for: Survival‐Assured Liver Injury Preconditioning (SALIC) Enables Robust Expansion of Human Hepatocytes in Fah –/– Rag2 –/– IL2rg –/– Rats
Source: Adv Sci (Weinh). 2021 Aug 11;8(19):2101188. doi: 10.1002/advs.202101188 (PMC8498896; doi:10.1002/advs.202101188)
Supplement: Supplementary file 1 — Supporting Information [file ADVS-8-2101188-s001.pdf]

© 2021 Wiley-VCH GmbH

ADVANCED SCIENCE Open Access

Supporting Information

for *Adv. Sci.,* DOI: 10.1002/advs.202101188

Survival-Assured Liver Injury Pre-Conditioning (SALIC) enables robust expansion of human hepatocytes in Fah$^{-/-}$Rag2$^{-/-}$IL2rg$^{-/-}$ rats

Ludi Zhang, Jian-Yun Ge, Yun-Wen Zheng, Zhen Sun, Chenhua Wang, Zhaoliang Peng, Baihua Wu, Mei Fang, Kinji Furuya, Xiaolong Ma, Yanjiao Shao, Nobuhiro Ohkohchi, Tatsuya Oda, Jianglin Fan, Guoyu Pan, Dali Li,* Lijian Hui**

Supporting Information

Survival-Assured Liver Injury Pre-Conditioning (SALIC) enables robust expansion of human hepatocytes in *Fah*$^{-/-}$*Rag2*$^{-/-}$*IL2rg*$^{-/-}$ rats

Ludi Zhang, Jian-Yun Ge, Yun-Wen Zheng, Zhen Sun, Chenhua Wang, Zhaoliang Peng, Baihua Wu, Mei Fang, Kinji Furuya, Xiaolong Ma, Yanjiao Shao, Nobuhiro Ohkohchi, Tatsuya Oda, Jianglin Fan, Guoyu Pan, Dali Li,* Lijian Hui**

Supplementary Figures

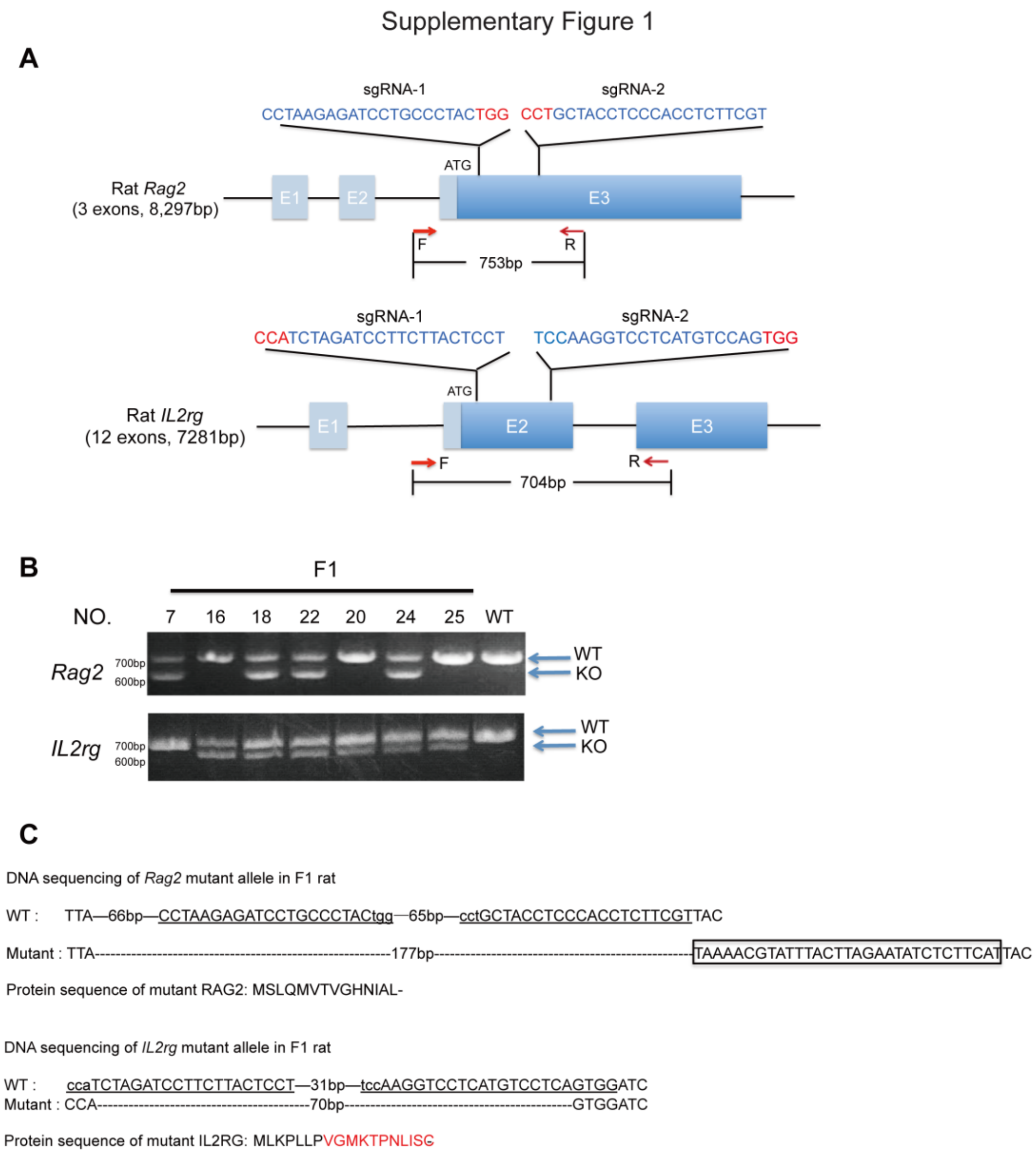

Figure S1. Generation of *Rag2*$^{-/-}$*IL2rg*$^{-/-}$ rats

(A) Schematic representation of the targeted exon of the rat Rag2 and IL2rg genes. The magnified views illustrate the binding sites of the sgRNAs. F and R represent the forward and reverse primers used for genotyping.

(B) The genotypes of the F_1 rats were determined by PCR analysis.

(C) The DNA and protein sequences of the mutant Rag2 and IL2rg genes in F_1 rats.

The amino acids in red, caused by frame-shifting mutations, were different from those in the wild-type protein sequence.

Supplementary Figure 2

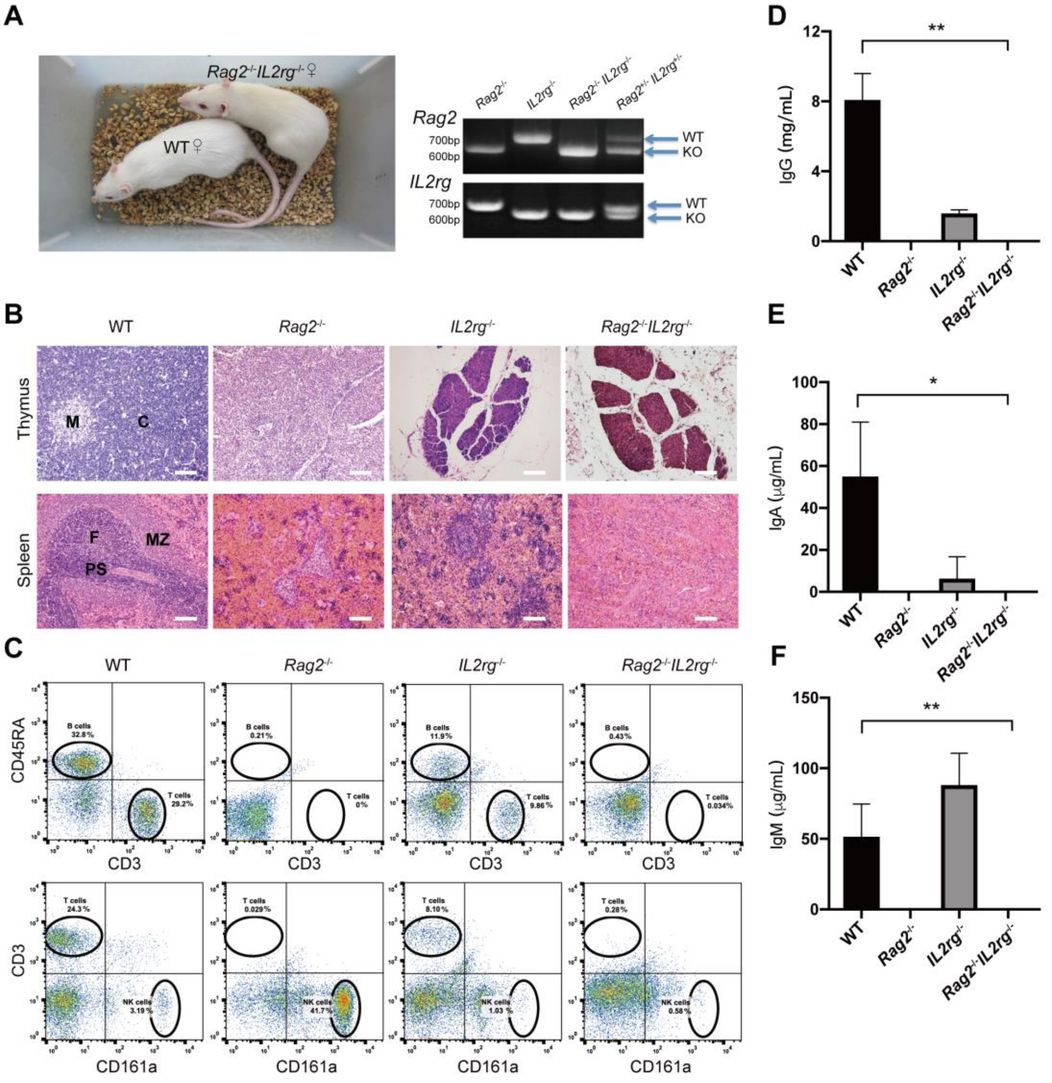

Figure S2. Characterization of severely immunodeficient *Rag2*$^{-/-}$*IL2rg*$^{-/-}$ rats

(A) Image and genotyping of *Rag2*$^{-/-}$*IL2rg*$^{-/-}$ rats.

(B) Histological analysis of the thymus and spleen from seven-week-old wild-type (WT), *Rag2*$^{-/-}$, *IL2rg*$^{-/-}$ and *Rag2*$^{-/-}$*IL2rg*$^{-/-}$ rats. WT thymus consists of cortex (C) and medulla (M) whereas the thymuses of *Rag2*$^{-/-}$*IL2rg*$^{-/-}$ rats were severely hypoplastic.

WT spleen consists of red pulp and white pulp with periarteriolar lymphoid sheaths (PS), lymphoid follicle (F), and marginal zone (MZ). In the spleens of *Rag2*$^{-/-}$*IL2rg*$^{-/-}$ rats, the white pulp was virtually devoid of lymphocytes. Scale bar, 100 µm.

(C) Flow cytometric analysis of T, B, and NK cell populations from spleens of WT, *Rag2*$^{-/-}$, *IL2rg*$^{-/-}$, and *Rag2*$^{-/-}$*IL2rg*$^{-/-}$ rats.

(D–F) Serum IgG (D), IgA (E), and IgM (F) levels in WT (n = 5), *Rag2*$^{-/-}$ (n = 2), *IL2rg*$^{-/-}$ (n = 3) and *Rag2*$^{-/-}$*IL2rg*$^{-/-}$ (n = 5) rats were determined by ELISA.

The data are shown as the mean ± SD. * $P < 0.05$, ** $P < 0.01$, Student's t test.

Supplementary Figure 3

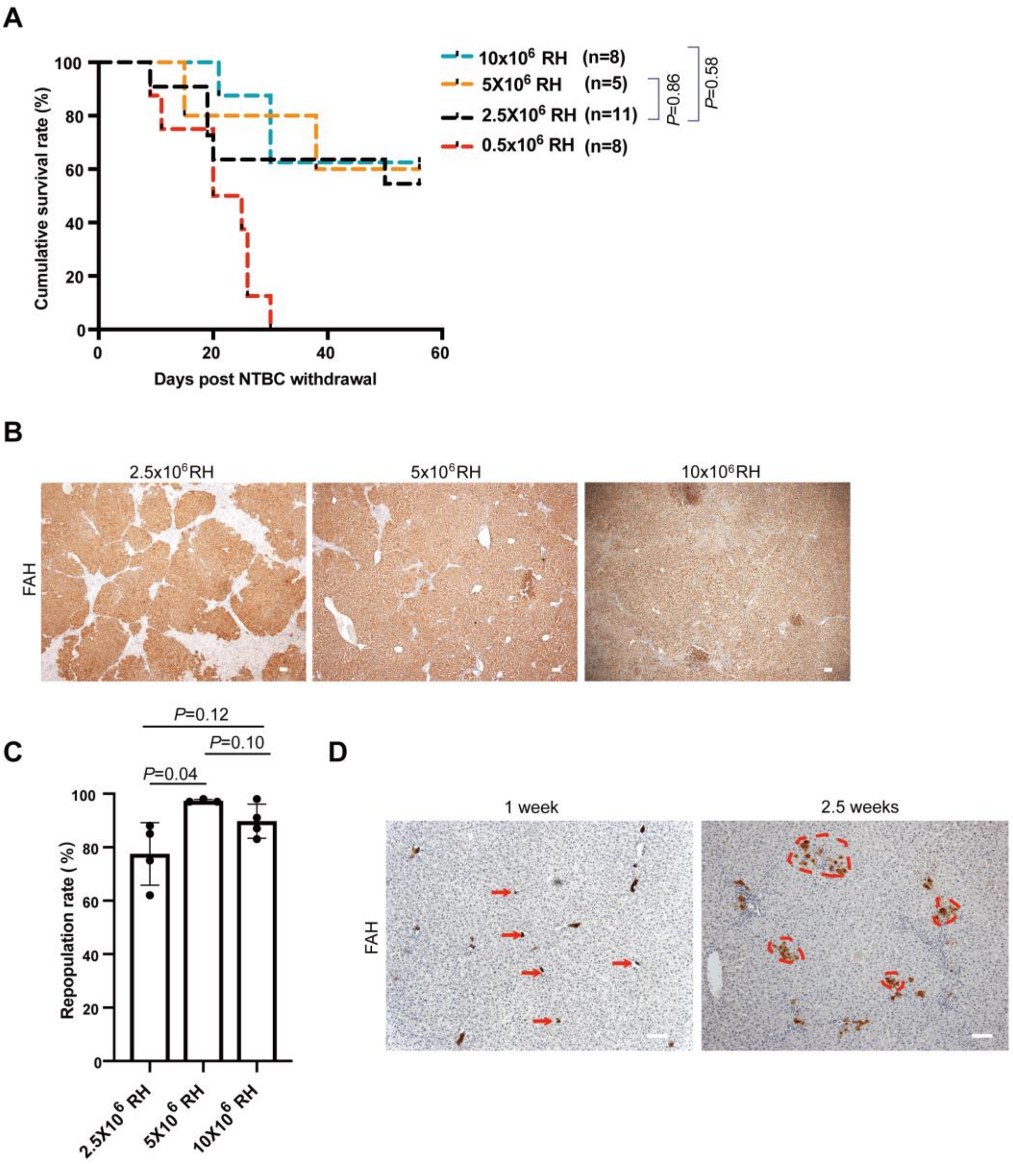

Figure S3. Transplantation of rat and mouse hepatocytes into *Fah*$^{-/-}$*Rag2*$^{-/-}$*IL2rg*$^{-/-}$ rats

(A) Kaplan–Meier survival curve of FRG rats transplanted with indicated cell doses of WT rat hepatocytes (RHs). $P > 0.05$, log-rank test.

(B–C) The repopulation rates were determined by FAH staining at 8 weeks after transplantation of different cell doses of RHs. Scale bar: 100 μm. The data are shown

as mean ± SD, Student's t test.

(D) The FAH staining of livers from moribund FRG rats transplanted with MHs without NTBC cycling. Scale bar, 100 μm.

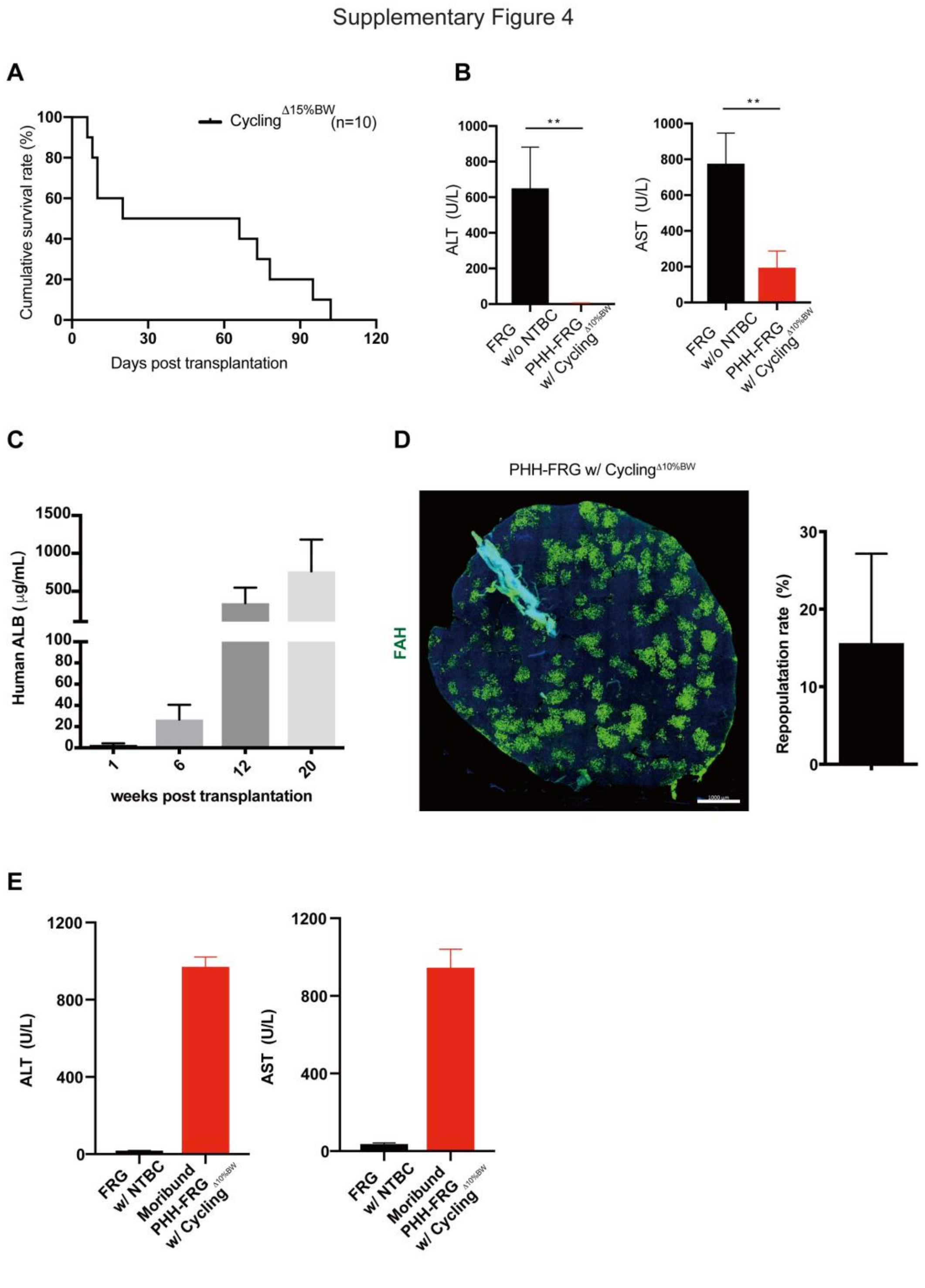

Figure S4. Pilot experiment for liver humanization under NTBC cycling$^{\Delta 10\%BW}$

(A) Kaplan–Meier survival curve of FRG rats transplanted with PHHs under NTBC cycling$^{\Delta 15\% BW}$.

(B) Serum ALT and AST levels in moribund FRG rats within 2 weeks after NTBC withdrawal (FRG w/o NTBC) and in PHH-transplanted FRG rats at 20 weeks after NTBC cycling$^{\Delta 10\%\ BW}$ (PHH-FRG w/ cycling$^{\Delta 10\%\ BW}$).

(C) The dynamic change of human ALB secretion level was monitored in the PHH-transplanted FRG rats with NTBC cycling$^{\Delta 10\%\ BW}$ for 20 weeks. n = 3.

(D) Representative FAH staining of PHH-FRG rat liver at 20 weeks. The repopulation rate was determined. n = 3. Scale bar, 1 mm.

(E) Liver function was determined in moribund FRG rats transplanted with PHHs under NTBC cycling $^{\Delta 10\%\ BW}$ for 8–12 weeks.

The data are shown as mean ± SD. ***P* < 0.01, Student's t test.

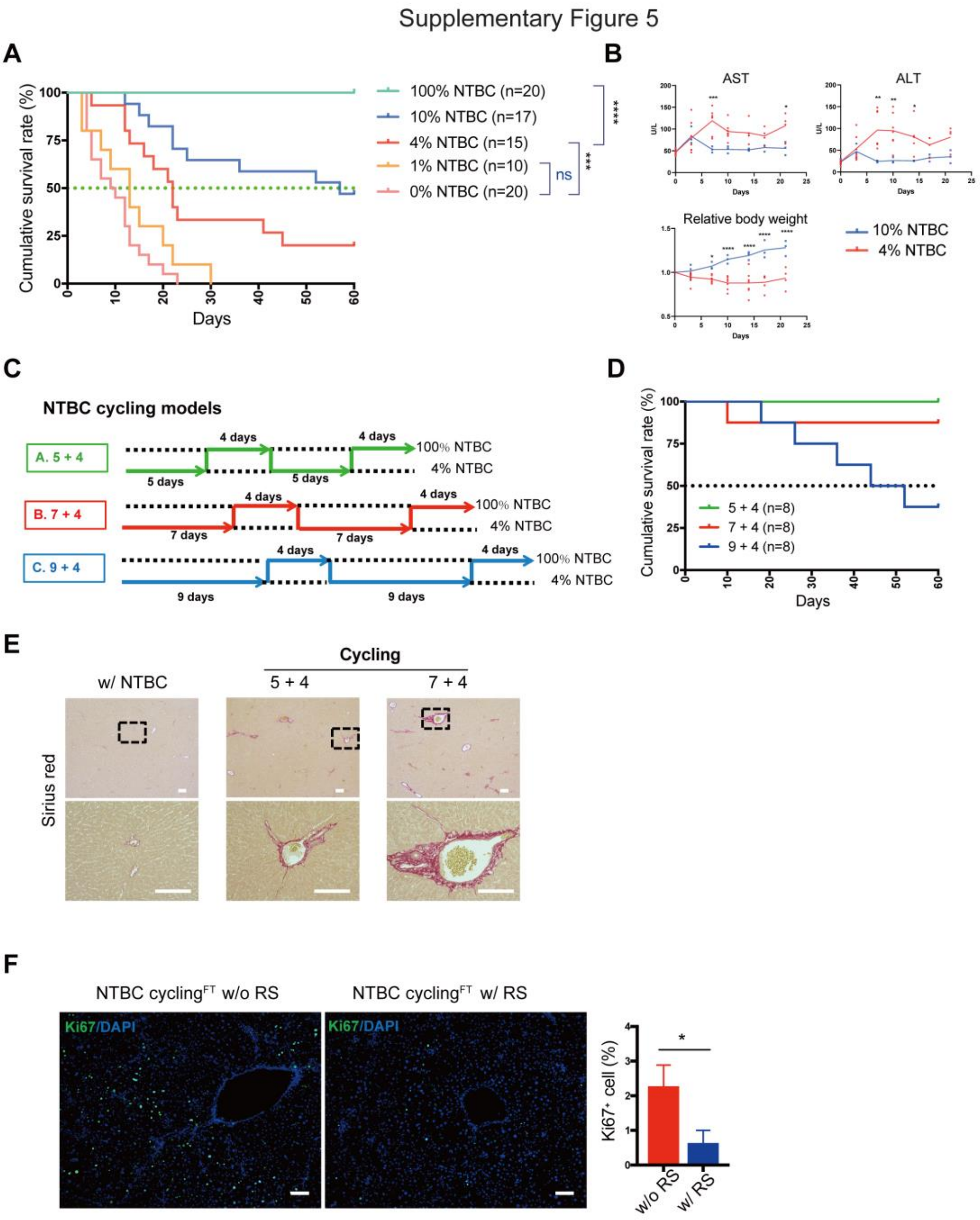

Figure S5. Establishment of survival-assured liver injury preconditioning

(A) Kaplan–Meier survival curve of FRG rats supplied with indicated concentrations of NTBC. 5 mg/L NTBC was defined as 100% NTBC.

(B) Levels of serum ALT and AST and relative bodyweight in FRG rats supplied with 10% (n = 3) and 4% (n = 7) NTBC for 3 weeks.

(C) Schematic outline of NTBC cycling models with defined time and concentrations.

Repeated administration with 4% NTBC for 5, 7, and 9 days to induce liver injury and subsequently 100% NTBC for 4 days (5+4, 7+4 and 9+4, respectively) to allow recovery was designed to induce liver injury.

(D–E) Kaplan–Meier survival curve (D) and liver fibrosis (E) of FRG rats under the treatment of different NTBC cycling models for two months. Scale bar, 100 µm.

(F) The proliferative cells in FRG rat livers were determined by Ki67 staining under NTBC cycling^{FT} for 2 months with or without retrorsine (RS) pre-conditioning. n = 3. Scale bar, 100 µm.

The data are shown as mean ± SD. ns $P > 0.05$, *$P < 0.05$, **$P < 0.01$, ***$P < 0.001$, ****$P < 0.001$, log-rank test for (A) and Student’s t test for (B, E, G, and H).

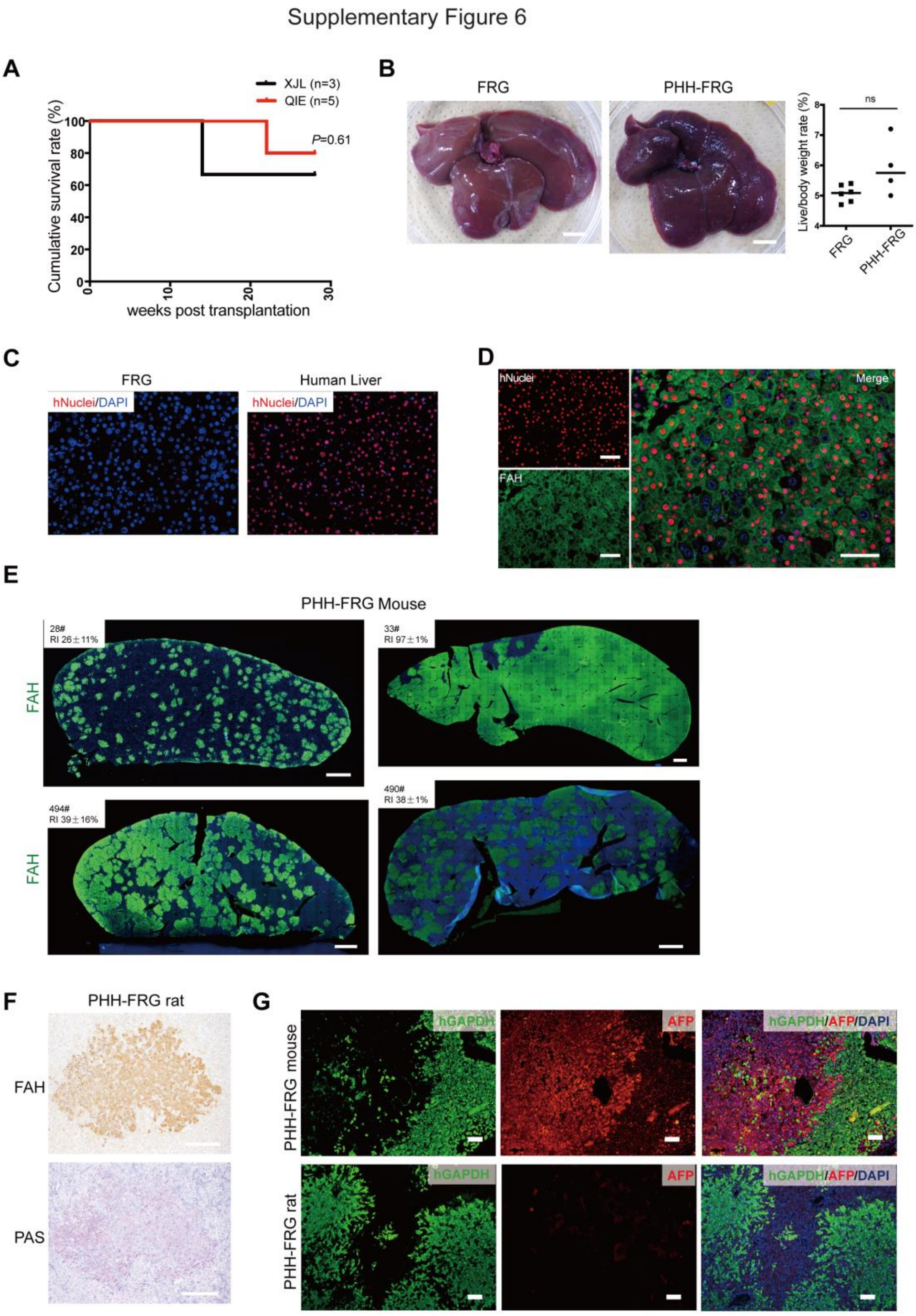

Figure S6. Characterization of PHH-repopulated FRG rats

(A) Kaplan–Meier survival curve of FRG rats transplanted with PHHs from two donors (XJL and QIE).

(B) Representative liver images and the liver/body weight ratios of the FRG rat with or without transplantation of PHHs at seven months.

(C) The specificity of the hNuclei antibody was determined by immunostaining of FRG rat liver and human liver.

(D) The hNuclei and FAH co-immunostaining of liver-humanized rat. Scale bar, 100 µm.

(E) The repopulation of PHHs in FRG mouse livers was determined by FAH staining seven months after transplantation. Scale bar, 1 mm.

(F) FAH and periodic acid–Schiff (PAS) staining of serial liver sections from PHH-FRG rats at seven months. Scale bar, 100 µm.

(G) AFP protein expression was examined with costaining of hGAPDH in PHH-repopulated FRG mouse and rat livers at 7 months. Scale bar: 100 µm.

The data are shown as the mean ± SD. ns $P > 0.05$, Student's t test for (A) and log-rank test for (D).

Supplementary Figure 7

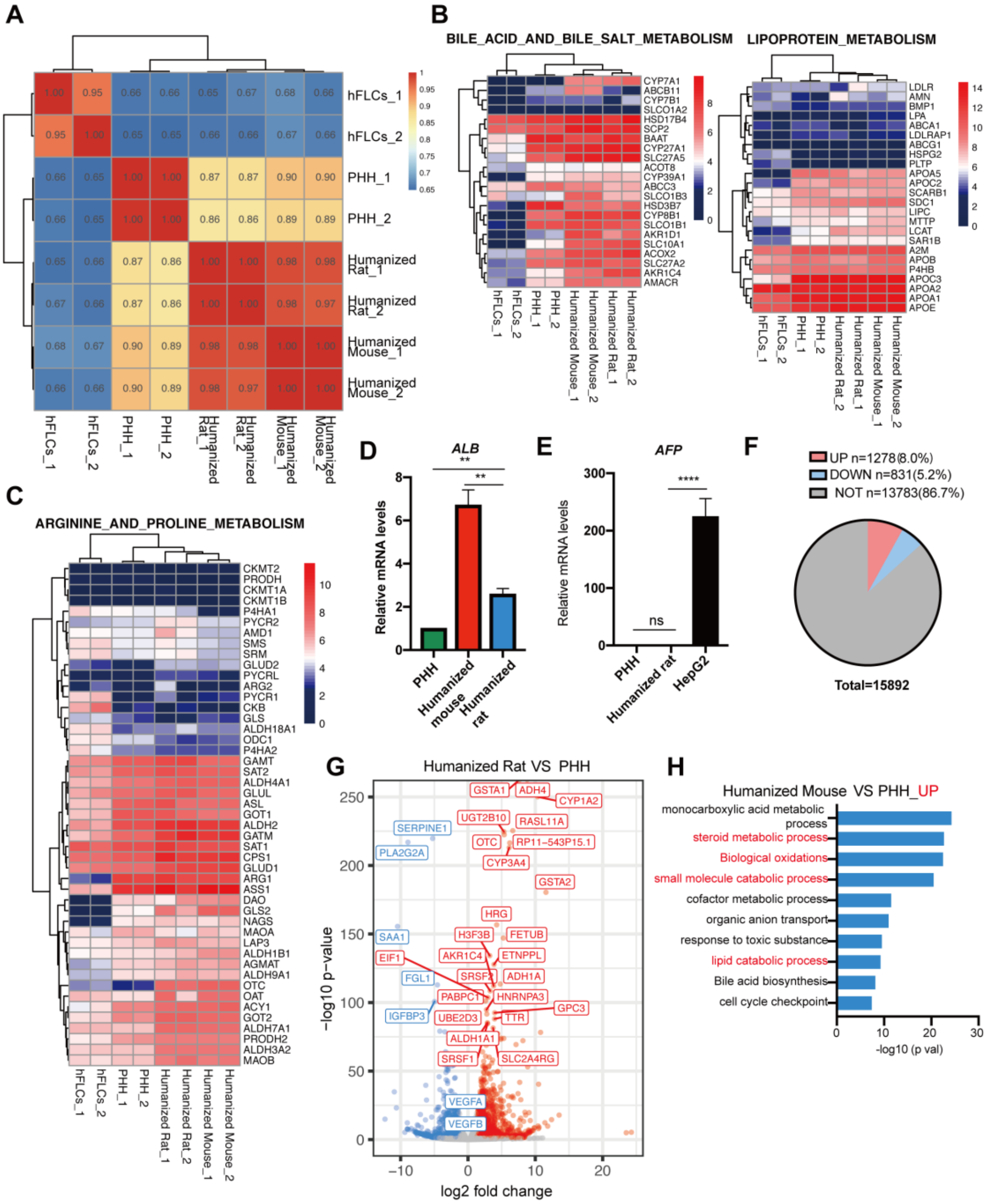

Figure S7. Transcriptome analysis of PHH-repopulated FRG rat livers

(A) Pearson correlation coefficient–based heat map representation of the similarity of gene expression profiles of human fetal liver cells (hFLCs), PHHs, and liver-humanized mouse (humanized mouse) and rat (humanized rat) livers. PHHs from the same donor were used.

(B–C) Heat map and hierarchical clustering of expression of genes involved in bile acid, lipoprotein (B), and arginine and proline (C) metabolism pathways from RNA-seq data of hFLCs, PHH, and liver-humanized mouse and rat livers.

(D–E) Comparison of gene expressions of human *ALB* (D) and *AFP* (E) in liver-humanized mouse (n = 3) and rat (n = 3) livers. Frozen PHH was used as positive control for ALB. HepG2 was used as positive control for AFP. Human-specific primers were used in qPCR. The data are shown as mean ± SD. ***P* < 0.01, *****P* < 0.0001, Student's t test.

(F–G) Differential expression analysis was performed between liver-humanized rat livers and PHH using DESeq2. Genes were considered differentially expressed if FPKM > 1 in all sample and fold changes ≥ 2.5, padj ≤ 0.05. The number of upregulated (UP) and downregulated genes (DOWN) was calculated.

(H) Differential expression analysis was performed between liver-humanized mouse livers and PHH using DESeq2. Gene ontology analysis was performed to identify enriched pathways in the upregulated genes (fold change ≥ 2.5) of liver-humanized mouse livers.

Supplementary Figure 8

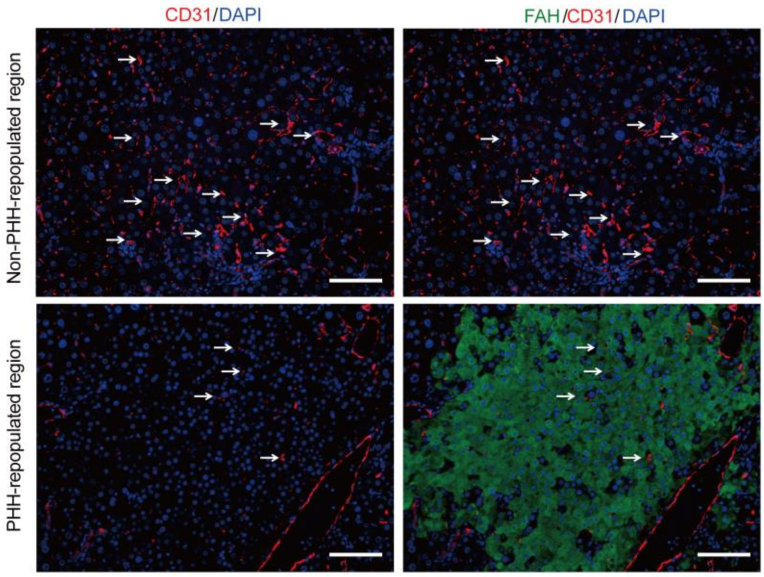

Figure S8. Reduced vessel density in the PHH-repopulated colony

Vessel density was determined by CD31 and FAH costaining in liver-humanized rats at 7 months after transplantation. Scale bar: 100 μm. White arrows indicate the vessels.

Supplementary Table 1

Drug	Group	Rat ID	T_{max} (h)	C_{max} (ng/ml)	$AUC_{(0\text{-}t)}$ (h*ng/ml)	$AUC_{(0\text{-}\infty)}$ (h*ng/ml)	$MRT_{(0\text{-}t)}$ (h)	$T_{1/2}$(h)
AZT	FRG rat	60	0.5	3613.9	5161.8	5234.8	1.5	1.2
		62	0.5	2394.6	3426.7	3475.5	1.6	1.3
		178	0.5	3580.5	3928.3	4011.0	1.1	0.7
		**mean**	**0.5**	**3196.4**	**4172.3**	**4240.4**	**1.4**	**1.0**
		**SD**	**0.0**	**694.5**	**892.9**	**901.8**	**0.3**	**0.3**
AZT	Humanized FRG rat	147	0.5	3135.6	4546.2	4585.6	1.5	1.1
		89	1	2944.6	7470.8	7511.3	1.7	1.1
		124	0.5	3102.1	3264.4	3275.9	1.3	0.9
		**mean**	**0.7**	**3060.8**	**5093.8**	**5124.2**	**1.5**	**1.0**
		**SD**	**0.3**	**102.0**	**2156.0**	**2168.5**	**0.2**	**0.1**
GAZT	FRG rat	60	0.5	221.7	251.9	260.7	1.1	0.8
		62	0.5	255.5	368.4	384.7	2.1	1.5
		178	0.5	189.7	199.7	206.8	1.2	0.7
		**mean**	**0.5**	**222.3**	**273.3**	**284.1**	**1.5**	**1.0**
		**SD**	**0.0**	**32.9**	**86.4**	**91.2**	**0.5**	**0.5**
GAZT	Humanized FRG rat	147	0.5	711.7	2327.5	2591.9	3.4	1.6
		89	1	898.1	3274.3	3402.6	2.4	1.6
		124	0.5	565.7	678.2	713.1	1.1	0.8
		**mean**	**0.7**	**725.1**	**2093.3**	**2235.9**	**2.3**	**1.4**
		**SD**	**0.3**	**166.6**	**1313.8**	**1379.6**	**1.1**	**0.5**

Table S1. Pharmacokinetic parameters of AZT and GAZT after oral administration of AZT to rats

Supplementary Table 2

Gene	Forward(5'-3')	Reverse(5'-3')
CYP2A6	CAGCACTTCCTGAATGAG	AGGTGACTGGGAGGACTTGAGGC
CYP3A4	TTCAGCAAGAAGAACAAGGACAA	GGTTGAAGAAGTCCTCCTAAGC
CYP2C9	GCCTGCCCCATGCAGTGACC	CACAGCAGCCAGCCAGGCCAT
CYP2E1	CCCCAGCGGCACCATGTCTG	TGGGCCAACCGGGTGAAGGAA
SLCO1B1	TTGGAGGTGTTTTGACTGCTT	ACAAGTGGATAAGGTCGATGTTG
GSTA1	CTGCCCGTATGTCCACCTG	AGCTCCTCGACGTAGTAGAGA
UGT2B7	TCAGCCCTGGCCCAGATCCC	ACAGCTGCTCCCCTGGCCTT
GAPDH	CCACCTTTGACGCTGGG	CATACCAGGAAATGAGCTTGACA
ALB	GCCTTTGCTCAGTATCTT	AGGTTTGGGTTGTCATCT
AFP	ACTGAATCCAGAACACTGCA	TGCAGTCAATGCATCTTTCA
Rat Fah	CTGCTGCATTTAAGCTACCAC	CATAGAAGCCAGGATGAGTGT
Rat Rag2	TTCCCAGAAAGCAAAGCAAAG	GCATGTATGACCGTCCTCCAA
Rat IL2rg	AAAGGTGGTTGGGAATGAAGG	AAGGTCCATAGAAGTCAGCAAGA

Table S2. List of primers for genotyping and qPCR

Experimental Section

Materials and Methods

FRG mice

The FRG mice were obtained from Dr. Xin Wang's Lab. FRG mice are on a hybrid strain of C57BL/6J and 129S6/ SvEvTac and fed with drinking water containing 7.5 mg/L NTBC. All animals were housed in a temperature- and light-controlled (12-h light/dark cycle) specific pathogen–free (SPF) animal facility, in individually ventilated cages always with companion animals.

Flow cytometric analysis of immune cell populations

Flow cytometric analysis of immune cell populations isolated from the thymus and spleen were carried out using Rat T/B/NK Cell Cocktail (CD3-APC/CD45RA-FTIC/CD161a-PE, BD Biosciences) and Rat Compensation Set (CD3-APC/CD4-PE/CD8 –FITC, BD Biosciences) according to the manufacturer's instructions.

Rat immunoglobulin ELISA

Serum was collected from WT, *Rag2*$^{-/-}$, *IL2rg*$^{-/-}$, and *Rag2*$^{-/-}$*IL2rg*$^{-/-}$ rats. Levels of rat IgA, IgG, and IgM were measured using rat IgA, IgG, and IgM ELISA Ready-SET-Go!® (ebioscience) according to the manufacturer's instructions. Serum was diluted in a 10- to 10,000-fold range to obtain values falling within the linear range of the standard curve.

Syngeneic hepatocyte transplantation in FRG rats

Six- to eight-week-old rats were subjected to standard two-step collagenase perfusion for isolation of primary hepatocytes. Viability of isolated hepatocytes was around 90% as determined by Trypan blue. 0.5×10^6, 2.5×10^6, 5×10^6, and 1×10^7 hepatocytes from WT littermates were respectively transplanted through the portal vein into FRG rats. NTBC was discontinued immediately after transplantation and all throughout the experiment. They were sacrificed eight weeks after transplantation.

Xenotransplantation of mouse hepatocytes in FRG rats

Six- to eight-week-old WT mice on a strain of C57BL/6J were purchased from Shanghai LinChang Biotech. Mice were subjected to standard two-step collagenase perfusion for isolation of primary hepatocytes. Viability of isolated hepatocytes was around 90% as determined by Trypan blue. 2.5×10^6 hepatocytes were transplanted through the portal vein into FRG rats. NTBC was discontinued immediately after transplantation, and body weight was monitored every two days. When rats lost over 15% of their body weight, NTBC was transiently put on for 4 days. They were sacrificed 8 weeks after transplantation. Liver and blood samples were collected immediately for further analyses.

Xenotransplantation of human hepatocytes in FRG mice

Six days before cell transplantation, concentration of NTBC in drinking water

for FRG mice was first reduced to 3.75 mg/L for 3 days and was then totally withdrawn for another 3 days. 1 × 10^6 PHH in 100 μL PBS were intrasplenically transplanted into FRG mice. After transplantation, NTBC was transiently put on for 4 days when mice lost over 15% of their body weight. Mice were sacrificed 7 months later.

Histology, immunohistochemistry, and immunofluorescence

Tissue samples were fixed overnight in 4% neutral-buffered paraformaldehyde (Solarbio), embedded in paraffin, cut into 5-micrometer-thick sections, and placed on adhesion microscope slides. Sections were subjected to hematoxylin-eosin (H&E) and Sirius red staining according to standard protocols. For immunohistochemistry, deparaffinized and rehydrated slides were subjected to autoclave antigen retrieval in a 10 mmol/L citric acid buffer (pH 6.0) and allowed to cool to room temperature. Slides were blocked with 3% H_2O_2 for 30 minutes, washed in phosphate-buffered saline (PBS), and blocked with 5% normal donkey serum in PBS. Slides were incubated with diluted primary antibodies overnight at 4°C. IHC staining was performed using the Elite ABC kit (Vector Laboratories) according to the manufacturer's protocol. For immunofluorescence, deparaffinized and rehydrated slides were washed 2 × 15 min in PBS and blocked in 5% normal donkey serum for 1 hour and stained with primary antibody overnight. OpalTM 4-Color Manual IHC Kit (PerkinElmer) was used according to the manufacturer's protocol.

For frozen sections, cryostat sections (7 μm) were fixed in 4% PFA for 10 min at room temperature and then blocked with 10% normal goat or donkey serum (Thermo Fisher Scientific) for 60 min at room temperature. The primary antibodies were incubated at 4°C overnight. Primary antibodies were detected using fluorescent-conjected second antibodies (Jackson Lab). Sections were stained with DAPI (4’,6-Diamidino-2-Phenylindole) and mounted with fluorescence mounting medium (Dako).

Antibodies used for staining are as follows: goat anti-human-albumin (Bethyl Laboratories, 1:1000), mouse anti-hNuclei (Millipore, 1:100), rabbit anti-AAT (NeoMarkers, 1:200), rabbit anti-FAH (Cell Lab Tech, 1:3000), rabbit anti-Ki67 (Abcam, 1:250), mouse anti-human CYP3A4 (Santa Cruz, 1:200), mouse anti-GS (BD, 1:200), rabbit anti-CYP1A2 (Bio-rad, 1:200), mouse anti-CK19 (Progen, 1:100), mouse anti-MRP2 (Genetex, 1:200), rabbit anti-ARG1 (Genetex, 1:200), rabbit anti-UGT2B7 (ThermoFisher, 1:200), rabbit anti-AFP (Dako, 1:100), Cy5-conjugated donkey anti-goat IgG (Jackson Lab, 1:500), FITC-conjugated donkey anti-rabbit IgG (Jackson Lab, 1:500), Cy3-conjugated donkey anti-rabbit IgG (Jackson Lab, 1:500), Cy3-conjugated donkey anti-mouse IgG (Jackson Lab, 1:1000), FITC-conjugated donkey anti-mouse IgG (Jackson Lab, 1:1000), Alexa Fluor 488-conjugated goat anti-mouse IgG1 (Thermo Fisher Scientific, 1:500), and Alexa Fluor 647-conjugated goat anti-mouse IgG2b (Thermo Fisher Scientific, 1:500).

PAS staining

Paraffin sections were deparaffinized, rehydrated, and then stained by periodic acid–Schiff (Shanghai yuanye Bio-Technology) following manufacturer's instructions.

qPCR

For most experiments, total RNA was isolated from liver tissue by Trizol (Invitrogen). RNA extracted from freshly thawed PHHs was used as controls. 1 μg RNA was reverse-transcribed into cDNA with M-MLV Reverse Transcriptase (Promega) according to manufacturer's instructions. PCR was performed with HiFi Taq polymerase (TransGen). Quantitative real-time PCR was performed with SYBR Premix Ex Taq (TaKaRa) on ABI StepOnePlus real-time PCR system (Applied Biosystems). All q-PCR data were performed with at least two repeats. Primer sequences are provided in Table S2.
